# Does Abortion Liberalisation Accelerate Fertility Decline? A Worldwide Time-Series Analysis

**DOI:** 10.1007/s10680-023-09687-y

**Published:** 2023-12-05

**Authors:** Juan J. Fernández, Dácil Juif

**Affiliations:** Universidad Carlos III of Madrid, Madrid, Spain

**Keywords:** Abortion policy, Fertility rate, Time-series

## Abstract

Since WWII, the two most important global trends in family planning have been fertility decline and abortion liberalisation. But are they related? Specifically: Does abortion liberalisation affect changes in fertility rates? The demographic literature has yet to answer this important question and instead offers two opposing predictions. Some studies argue that liberalisation of this medical procedure reduces fertility rates. By contrast, others note that such legal reforms may merely have an average, negligible effect on fertility levels. We adjudicate between the two approaches by conducting, in our view, the most comprehensive global, quantitative analysis of the relationship between those legal reforms and changing fertility rates. The analysis relies on two-way fixed models and three different indicators of abortion policy liberalism created by independent research teams to estimate the relationship between abortion liberalisation and total fertility changes. The data cover 185 independent states between 1970 and 2019. Fertility rates are significantly related to average public education levels and alternative contraceptive use. Using multiple model specifications, however, abortion reforms do not have a robust association with the outcome. Replication materials for this article can be found at https://figshare.com/s/5336a4422f47c8c39228.

**Supplementary Information:**

The online version contains supplementary material available at 10.1007/s10680-023-09687-y.

What is the association between abortion liberalisation law and national fertility levels? Since the end of WWII, many governments have passed abortion reforms as a means of controlling fertility (Robinson & Ross, 2007). In many cases, the policy rationale behind abortion law reforms was to improve women’s health and legal rights (Clarke & Mühlrad, 2021; Cook & Dickens, 2003). However, China in the 1950s, and India in the 1970s, passed abortion liberalisation laws as part of family planning policies (Hirve, 2004; Wang, 2012). One aim of these policies was to control fast population growth, which was deemed to undermine economic growth. With the exact opposite objective, in 1966 Romania’s government passed a partial abortion decriminalisation law to raise the country’s plummeting fertility rate (Teitelbaum, 1972).

Although the literature on abortion liberalisation’s impact on fertility rates is already sizeable, it fails to clarify whether abortion reforms, *on average*, substantially affect fertility. This stems from the literature relying mainly on case-studies (González et al., 2018; Levine et al., 1999; Pop-Eleches, 2010) that cover a limited range of potentially non-representative reforms. Additionally, a single cross-national, time-series study by Bloom et al. (2009) on the determinants of female labour force participation reported comparative evidence of the reform-fertility link, but only as part of a subsidiary analysis. We therefore have a surprisingly limited understanding of the association between abortion liberalisation and the well-known, worldwide fertility decline that took place after WWII (Boyle et al., 2015; Bryant, 2007; Fernández, 2021; Forman-Rabinovici & Sommer, 2018; United Nations, 2019). The 2022 overturning of nationwide abortion rights in the USA (Dobbs v. Jackson Women's Health Organization ruling) has further reinforced the need for comparative analyses of the repercussions of abortion reforms. This ruling indicates very clearly that the status of women’s rights in many countries is fragile and that a better understanding of the past link between abortion reform and fertility could shed light on the future link between further re-criminalisation and fertility changes.

This article provides the most comprehensive, truly global analysis of abortion reform’s influence on fertility rates. To ensure the robustness of the findings, we draw on three indicators of abortion policy liberalisation constructed by three different research teams (Fernández, 2021; Finlay et al., 2013; Forman-Rabinovici & Sommer, 2018). We also employ two-way fixed effects (FE) models to guarantee that the covariates of interest do not absorb the influence of country-specific characteristics or global trends.

Based on limited evidence and the widespread assumption that state policies can impinge on individual reproductive decisions, many empirical studies argue that abortion liberalisation accelerates a country’s demographic transition by reducing fertility rates (Bloom et al., 2009; Clarke & Mühlrad, 2016; Pop-Eleches, 2010). However, there are strong reasons to believe that abortion reforms may not actually have an average, negative association with the number of births an average woman has: e.g. in the absence of policy instruments to facilitate their implementation, *de jure* reforms may not translate into higher access to this form of birth control; other reliable methods of birth control may already be widely used; or abortion reform could even have the unintended influence of fostering risky sexual behaviour that increases the number of undesired pregnancies (Assifi et al., 2016; Canning, 2011; Klerman, 1999).

In support of the latter position of a demographic null association, the evidence presented below suggests that abortion liberalisation is not robustly associated with fertility changes. Using three different indicators, with and without socio-economic control variables and with and without imputation of missing values, we show that, on average, countries that decriminalise abortion do not tend to experience a faster decline in total fertility rates. This finding has important social and policy implications. It implies that concerns about the detrimental demographic effect of abortion liberalisation in ageing populations have been overstated (Reuters, 2017). Perhaps more importantly, it suggests that the decriminalisation of abortion constitutes an ineffective means of harnessing a demographic dividend in high-fertility countries.

## Fertility Effects of Abortion Law Reforms

We define abortion here as the induced termination of a pregnancy by medical or surgical means. It thereby differs from miscarriage, which refers to the spontaneous termination of a pregnancy. Induced abortion—hereafter, abortion—is a common method of deliberate birth control, both in countries where its practice is legally permitted and countries where it is not—in the latter case, seriously compromising many pregnant women’s health (Grimes, 2006; Johnson et al., 2017; Latt et al., 2019). The practice of induced abortion has been deemed a potential determinant of fertility reduction at least since John Bongaarts’ (1978) famous work on “The Proximate Determinants of Fertility”, as the termination of unwanted pregnancies is assumed to result in a lower total number of births (Davis & Blake, 1956). More importantly, the link between abortion law reform and fertility changes has been directly or indirectly addressed by extensive literature on demography and public health. However, this literature advances two opposing positions: (1) abortion liberalisation reduces fertility levels or (2) has a null effect over them. This section summarises the theoretical reasoning behind the two positions before reviewing the findings of empirical research.

### The Case for a Negative Impact of Abortion Liberalisation on Fertility Changes

The reasons for a potential negative effect of abortion liberalisation on fertility changes are rather intuitive. They were most explicitly formulated by Levine (2005). Levine conceptualised the decision to have an abortion from a rational-action perspective and as a matter of relative personal costs. If the pregnancy is unwanted, women or couples decide whether to terminate a pregnancy by balancing the costs of an abortion against the costs of an unwanted birth. They will decide to have an abortion if its overall costs are lower than those of continuing with the unwanted pregnancy. As far as Levine was concerned, the legal status of this medical procedure heavily impinges on the costs incurred. If the procedure is illegal, women could suffer dire penal repercussions, increasing the costs of this option and making it rarely worthwhile. However, if this procedure is legal and the penal liabilities disappear, the costs will be merely economic and can easily undercut those of carrying on with an unwanted pregnancy.

Applying the model to the USA, Levine (2005, p. 56) argued that “its widespread legalisation in the early 1970s led to a very large reduction in its cost, and for many women the cost of abortion fell below the cost of an unwanted birth. This would support the prediction that abortion legalisation reduced unwanted births”. In discussing the consequences of abortion and other fertility regulation technologies, Potts (1997, p. 1) argued, in similar terms, that “human beings have been able to exercise conscious control over their fertility since the second half of the twentieth century, but wherever access to birth control technologies is not constrained by *law* [emphasis added], policies, custom or economic factors, there has been a marked fall in family size”. The expectation of a negative association has also percolated into reviews of regional demographic change (Cleland et al., 2006) and those produced by international organisations. The United Nation’s (2014, p. 14) report on *Abortion Policies and Reproductive Health around the World* noted that “restrictive abortion policies may contribute directly to higher fertility rate levels by reducing the probability of terminating an unwanted pregnancy”.

In other words, a widely shared assumption in demography states that since abortion liberalisation removes the legal sanctions for performing this medical procedure, as well as the personal costs to access this method of birth control, it results in a reduction in unwanted births and, therefore, the total number of births. This expectation of a link between abortion legality and demographic trends has been heavily present in policy-making circles since at least the 1950s. Indeed, over the last seven decades many countries have passed abortion reforms as an integral part of their population policies and as an instrument to increase or reduce population growth (Guillaume et al., 2018). Based on this reasoning, we hypothesise that abortion decriminalisation reduces the fertility rate (H1).

### The Case for a Null Impact of Abortion Liberalisation on Fertility Changes

Despite the intuitiveness of a potential influence of abortion liberalisation on fertility, there are also reasons why these two dimensions may not be causally related. First, *de jure* changes may not necessarily guarantee *access* to this method of birth control. Access to de facto abortion can be severely restricted by a limited supply of family planning centres—particularly in jurisdictions where the procedure may only be performed in hospitals and clinics where conscientious objection by doctors on religious, moral and social grounds is prevalent; by persistent stigmatisation of this medical procedure; and by limited awareness among women of abortion’s legal status (Assifi et al., 2016; Githens & McBride Stetson, 1996; Singh et al., 2018). There are also substantial differences between countries as to how courts and medical committees have interpreted legal rights to abortion (Davis & Blake, 1956).

Second, induced abortion is but one way of achieving fertility control. Whether or not abortion is legal, women and couples may alternatively decide to rely on other contraceptive technologies to achieve their desired family size. One example is the Netherlands, a country where abortion on request has been legal for five decades, but is today a “relatively rare phenomenon” (Levels et al., 2012, p. 302). Instead of relying on induced abortion, Dutch women rely on less invasive contraceptive pills. Likewise, in many countries where abortion remains largely illegal, other forms of contraception may in fact be legal. Therefore, in the absence of legally sanctioned abortion, women and couples could, if available, resort to other contraceptive technologies to prevent unwanted births. In this sense, Canning (2011, p. 355) holds that “the availability of contraception and abortion clearly affects fertility rates, but may not be decisive in allowing fertility to decline”.

Third, abortion liberalisation may have unintended consequences: it may influence the use of other contraceptives and sexual activity (Levine & Staiger, 2004). By inciting more people to engage in unprotected and risky sexual behaviour (Klick et al., 2012), it could result in more pregnancies—not all of them ending in abortions. According to this approach, abortion liberalisation does not therefore reduce the fertility rate on average (David, 1992). Following this reasoning, we hypothesise that abortion liberalisation has a null impact on fertility changes (H2).

### Empirical Research on the Link Between Abortion Reform and Fertility Changes

Several empirical works on abortion policy-fertility links provide interesting tests of a potential causal effect. These quantitative studies—where case studies predominate—display largely consistent patterns and document that abortion decriminalisation tends to occur hand in hand with substantial reductions in fertility. Among the first states to decriminalise abortion were the former communist countries in Eastern Europe. In the absence of other forms of family planning, abortion was deemed a necessary evil to facilitate industrialisation and resulted in socialist countries adopting very liberal abortion laws after Stalin’s death (Bradatan & Firebaugh, 2007; Githens & McBride Stetson, 1996). Accordingly, Romania decriminalised abortion in 1957. However, soon after the communist regime performed a drastic policy turnaround, re-criminalising abortion almost completely and banning the production of contraceptives to promote population growth. In an early analysis of this outlier case, Pop-Eleches (2010) later found that the effect of the abortion ban on fertility in Romania was longer-lasting, as women who spent most of their reproductive years under the 23-year, pro-natalist regime (1966 to 1989) experienced increases in lifecycle fertility of about 0.5 children (see also Teitelbaum, 1972). Following a comparison of birth rates in Bulgaria, Romania and Poland before and after abortion reforms, Levine (2005: 157) concluded that “legalising abortion reduces births, whereas moderate restrictions on abortion within a legal abortion environment reduce pregnancies”.

Most of the recent empirical research on the link between abortion reform and fertility changes has, however, focussed on the United States (Bailey & Lindo, 2018). Scholars have used the liberalisation of abortion laws in the USA in the 1960s and 1970s and the subsequent implementation of restrictions on abortion access as natural experiments to analyse the short-term effects of abortion legalisation on fertility levels (Bailey & Lindo, 2018). For example, by comparing states that had and had not liberalised abortion before *Roe versus Wade,* Levine et al. (1999) showed in a quasi-experimental, difference-in-difference design that the nationwide legalisation of abortion by the Supreme Court’s 1973 *Roe versus Wade* decision led to a *circa* 4% reduction in the birth rate (Levine et al., 1996; Levine, 2005; Ananat, 2007).

Other studies have assessed the effects of post-*Roe vs. Wade,* state-level policy reforms such as the introduction of Medicaid abortion funding restrictions on de facto* access to* abortion (Klerman, 1999; Levine et al., 1996). Most of these studies found that the loss of Medicaid funding for abortions and the reduction in abortion providers have had a substantial negative effect on abortion incidence (Bearak et al., 2017; Fischer et al., 2018; Lindo et al., 2020). Legal restrictions on abortion access have also had a positive effect on births (Fischer et al., 2018; Guldi, 2008).

Regarding Western European countries, González et al. (2018) analysed the 1985 partial legalisation of abortion in Spain and found that the abortion law reform led to a decrease in the number of births among women aged 21 and younger. Levels et al. (2012) tested for policy effects using microdata for the Netherlands and showed that abortions of unintended pregnancies were significantly more likely in the post-1971 period when the medical procedure became legal in the country. Mølland (2016), using difference-in-difference analyses, found that abortion availability in Oslo in the 1960s delayed fertility but did not reduce the completed family size. Additionally, it resulted in higher educational attainment among mothers and children of mothers who had access to abortion.

Scholars have also leveraged recent abortion reforms in developing countries to further explore the policy-fertility link. Mexico City legalised abortion on request in 2007 and Clarke and Mühlrad (2016) also used difference-in-difference methods to examine the fertility and maternal health effect of this reform. Their results suggest that this legislation resulted in a reduction in births for several age groups (Gutiérrez Vázquez & Parrado, 2016). The case of Nepal has also attracted scholarly attention. After decades of no abortion reform, in 2002 Nepal legalised abortion on request and the country then saw a drastic fall in the fertility rate from 4.1 in 2001 to 2.6 in 2011 that Henderson et al. (2013) attributed to this reform.

The non-negligible number of case studies on abortion policy effects has not been paralleled by large-N comparative studies. In a study on the determinants of female labour force participation, Bloom et al. (2009) used abortion decriminalisation as an instrument of the fertility rate to assess the latter’s effect on female labour force participation. Using two-way fixed-effects, they found that changes in the abortion index had an average negative and significant effect on fertility. The maximum increase in the abortion index (from 0 to 7 legal conditions) leads to a predicted reduction in fertility of about 0.4 children. More recently, Bearak et al. (2020) utilised complex imputation models to estimate unintended pregnancy and abortion rates worldwide in 1990–1994 and 2015–2019. They showed that in 2015–2019 abortion rates did not differ significantly in each legal regime. In 1990–1994, however, countries with largely legal abortion had statistically higher abortion rates than countries where abortion was restricted.

While previous research provides helpful evidence of the link between abortion reform and fertility changes, it has two limitations. First, most studies involve case studies of only a few reforms that may not be representative of standard abortion decriminalisation. Moreover, persistent publication bias might mean that case studies reporting null findings (Antón et al., 2016) could be underrepresented. Second, the study by Bloom et al. (2009) examined the decriminalisation-fertility link in only 97 countries, which means that their results may differ if a larger sample were considered. Hence we still have a limited understanding of what the average, worldwide effect of abortion reform on fertility changes actually is.

## Data and Method

We contribute to the literature on the policy determinants of demographic trends through a longitudinal, worldwide analysis of the relationship between abortion law reform and fertility rates. Our study covers 195 countries through 50 years—1970 to 2019—to address the concrete question: Does the liberalisation of abortion laws have a direct association with the average fertility levels of most independent states?

Following convention in demographic research, the dependent variable in the analysis is the total fertility rate (TFR), i.e. the average number of children women are expected to bear through their fertile years given the current—age specific—birth rates. Formal definitions and the sources of the dependent variable and all independent variables are included in the Online Appendix.

Regarding the key independent variable—the *de jure* legal status of abortion—several databases on domestic abortion policy in most independent states have been recently published. Given the availability of multiple indicators, following Firebaugh (2007) this study maximises the robustness of the findings by drawing on three of these existing abortion policy indexes. *Abortion policy index 1* constitutes an expanded and updated version of the database Fernández (2021) constructed to assess the determinants of the liberalisation of abortion on request and for socio-economic reasons in 195 countries. This database includes six dichotomous variables identifying whether six legal grounds for conducting an abortion—life risks, health risks, rape, foetal impairment, socio-economic conditions and abortion on request—are legal (1) or illegal (0). Due to the high correlation between the six dichotomous items, we have summed these six conditions in *abortion policy index 1*.^1^

*Abortion policy index 2* draws on the abortion policy database constructed by Finlay et al. (2013) that covers 185 countries from 1960 to 2011 and considers the legal status of abortion under seven conditions: life risks of the pregnant woman, physical health risks, mental health risks, foetal impairment, rape, socio-economic grounds and abortion on request. *Abortion policy index 2* represents the sum of these seven legal grounds.

*Abortion policy index 3* draws on the database constructed by Forman-Rabinovici and Sommer (2018) that covers 193 countries from 1992 to 2015 and considers the legal status of abortion under the same seven conditions as Finlay et al. (2013). Unlike for Finlay et al. (2013), Forman-Rabinovici and Sommer’s preferred index is not the unweighted sum of all legal criteria. Rather, they weigh each legal criterion based on the proportion of countries where it is legal, so that a rarely legal criterion is given a higher value than a widely accepted criterion. The resulting *abortion policy index 3* thus sums the number of weighted criteria and is then rescaled to 0–1. Unsurprisingly, the three resulting indexes of abortion policy display high levels of correlation between *r* = 0.88 and *r* = 0.93 (Table A2).

In the considered period, several countries have regulated abortion at subnational level (e.g. Australia, Mexico, Nigeria or the United States). Assigning the abortion policy status of a region or state to the whole country would constitute an overgeneralisation and make estimates imprecise. All the following models are thus estimated without these countries.

In isolation, the abortion policy indexes could capture the influence of other conditions. In the models, we therefore control for other factors that have been proven or theorised to affect changes in fertility. We first draw on demographic theories explaining the fertility transition. Notestein’s (1953) and Thompson’s (1929) classic demographic transition theory attributes fertility declines to changes in social life that accompany industrialisation and urbanisation. In their theory, urbanisation increases the cost of raising children, while the rise in demand and the monetary returns on human capital that accompanies industrialisation triggers a shift in parents’ preference towards having fewer, better educated, children. The models capture this prediction through the *urban population* variable, which represents the percentage of population living in urban areas.

Using country-level evidence, Bloom et al. (2009) showed that female education is a significant predictor of the TFR, but male education is not. Following this reasoning, the models control for *average years of women’s education*. Beyond education, women’s formal, basic rights may also affect fertility decisions. If women attain stronger civil rights (private property, freedom of movement, freedom from forced labour and freedom to file a case in court) they may be more likely to control their fertility as well. *Women’s civil rights* controls for this potential factor. Since fertility may also depend on the economic empowerment of women, all models, furthermore, control for the *ratio of female to male labour force participation*.

*Infant mortality* is another well-established determinant of fertility decline. Davis and Blake (1956) argued that when mortality declines, parents realise that the probability of survival of all their offspring increases, and that they need to have fewer children to reach their desired number of adult children. The professionalisation of medicine in developing countries could also impinge on fertility levels as licensed physicians provide reliable recommendations on birth control methods. *Physicians per capita* captures this potential explanatory factor.

Economic crises increase the marginal cost of raising children and—at least for affluent democracies—have proven to produce immediate declines in birth rates. We therefore control for *GDP per capita*. Independently from structural and economic conditions, access to other methods of birth control also influences general fertility levels. The most obvious factor in this respect involves the use of other modern birth control methods, apart from induced abortion, hence we control for *contraceptive use*. Since data on contraceptive use is only available for 1970–2020, subsequent analyses all exclude events prior to 1970. ‘Abortion tourism’ (Linders, 2004) from a given country may hinder the impact of abortion liberalisation in that country on fertility levels. We therefore control for the *average value of the abortion policy index* in neighbouring countries.

Table A1 includes descriptive statistics of all non-imputed variables and indicates that several independent variables have a non-negligible number of missing values, which mainly belong to less developed and developing countries. We have therefore imputed missing values for all independent variables using multiple imputation (ten sets of imputations). Missing values for dependent variables were also imputed in the chained process, but only non-imputed information on the dependent variable was included in the final analyses.^2^ The three policy indices cover different periods: 1970–2019, 1970–2006 and 1992–2015 (*abortion policy index 1*, *abortion policy index 2* and *abortion policy index 3*, respectively). In the case of indices 2 and 3, using imputed values for the period not covered by the original dataset would make coefficient estimates excessively dependent on imputed and therefore uncertain values. In models including abortion policy indexes 2 and 3, we therefore restrict the analysis to the period covered by the two original databases. We use the logarithmic transformation of *GDP per capita, physicians per capita* and *infant mortality* because of their right-hand skew. Table A2 includes a correlation matrix of all variables.

Concerning the analytical strategy, for our preferred specification we run models with two-way fixed effects (TWFE) (Imai & Kim, 2021). A major advantage of TWFE is that country-FE absorb time invariant differences by country—e.g. pro-natality culture—that could be correlated with time varying covariates, whereas time dummies capture unobserved worldwide fertility trends. By following this strategy, we assess the association of an abortion reform with changes in fertility rates within countries that are independent of global downward fertility trends and country-specific characteristics. Equation (1) captures the functional form of the estimated models. In this equation, *y* is the outcome—the fertility rate—of country *j* and time *t*; *β*_0_ is the constant; *β*_w_ is the role of the abortion policy index; and *β*_x_ are the time-varying control variables. The equation also includes country-FE (${\beta }_{n} \,\text{country FE}_{j}$) and year-FE (${\beta }_{z}\,\text{year}_{t}$) while ${e}_{jt}$ represents the error term. In sensitivity models we also include linear time trends (through interactions between the country dummies and a linear time trend). However, we do not consider that to be our preferred specification, as a country-specific linear time trend may cause overfitting due to multicollinearity between the country-specific time trends themselves and slow-moving explanatory variables.

1 $${y}_{tj}={\beta }_{0}+{\beta }_{w}\text{ abortion policy index}_{jt}+{\beta }_{x} \text{ control variables}_{jt}+{\beta }_{z}\text{ year FE}_{t}+{\beta }_{n}\text{ country FE}_{j}+{e}_{jt}$$

## Results

We conduct the analysis in two stages. First, we assess general trends in the TFR and *abortion policy index 1*. Second, we assess the association between the three abortion policy indexes and fertility changes. In the latter cases, we consider the association between changes in the policy indexes and the outcome with and without control variables and with and without multiple imputation of missing values in the independent variables.

If most countries had not observed substantial changes in their fertility rates and had not revamped their abortion policy between 1970 and 2019, an analysis of the relationship between changes in both dimensions would be unjustified. However, the evidence points to substantial temporal shifts in both regards. Using the United Nations’ estimates for this period, the average annual change in the TFR was − 0.055 absolute points (*p* < 0.05). Moreover, this trend differed substantially across countries. Considering extreme cases of fertility change, Libya observed an absolute 5.56-point decline between 1970 and 2019, while the Central African Republic only had an absolute 0.006-point increase.

**Fig. 1:**
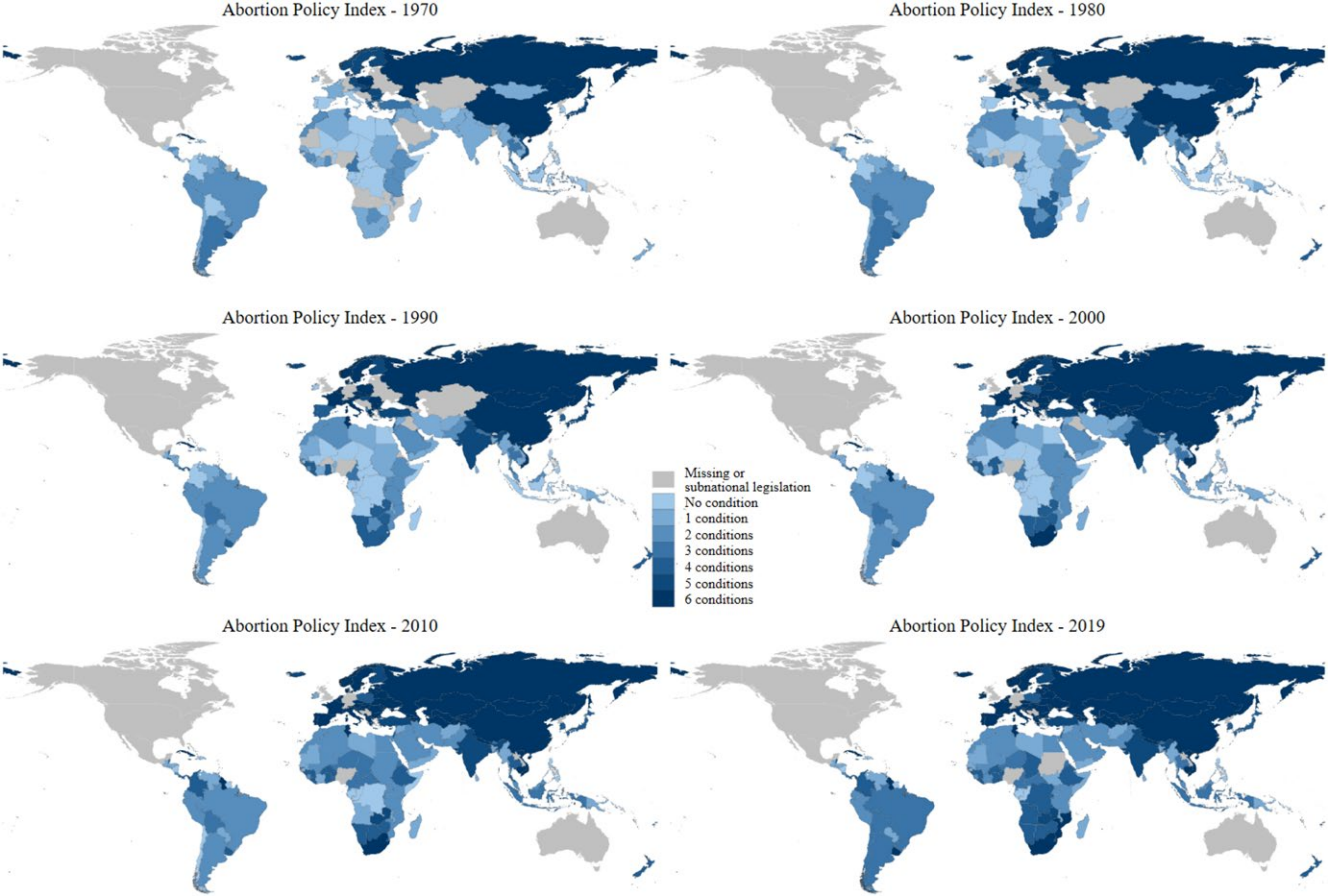
Abortion policy index, 1970–2019

Abortion policy has also observed major changes over these decades. Figure 1 depicts the average value in *abortion policy index 1* in all 195 countries considered at six time points: 1970, 1980, 1990, 2000, 2010 and 2019. Consistent with previous research (Bailey & Lindo, 2018; Boyle et al., 2015; Forman-Rabinovici & Sommer, 2018), Fig. 1 indicates a global secular trend of increasing abortion policy liberalism. In fact, the average *abortion policy index 1* increased from 1.71 in 1970 to 3.46 in 2019. This trend towards more liberal legal treatment of abortion also affected all six considered grounds: life risks, health risks, rape, foetal impairment, socio-economic grounds and abortion on request (Figure A1).

**Fig. 2:**
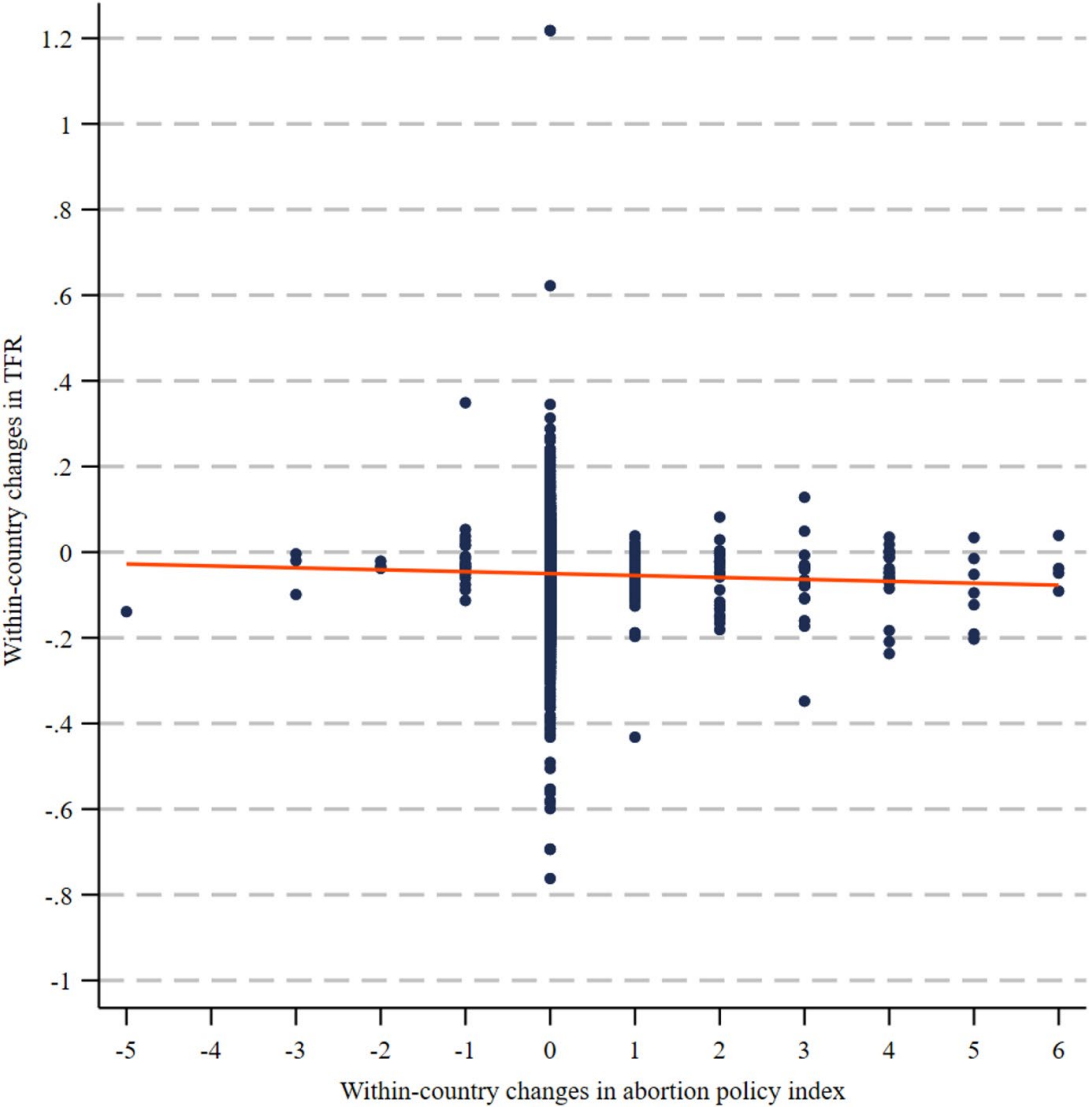
Relationship between within-country changes in TFR and within-country changes in abortion policy index, 1970–2019

The combined facts of cross-nationally variable trends in fertility decline and abortion policy reform warrant an analysis of the potential relationship between these two dimensions. Do countries that undergo abortion liberalisation display a steeper decline in the TFR? To provide preliminary evidence, Fig. 2 depicts the bivariate relationship between yearly changes in *abortion policy index 1* and yearly changes in the TFR as a negative relationship between both factors. According to the linear function, a one-point increase in yearly growth in the *abortion policy index* produces a meagre − 0.005 change in the TFR (*p* = 0.046). The above evidence does not indicate a strong association between abortion liberalisation and fertility changes.

However, this relationship may nevertheless be affected by global fertility trends. Using two-way FE models with *abortion policy index 1* as the key independent variable, Table 1 therefore conducts a more refined test of the unconditional influence of abortion reform. It includes 12 models that allow us to determine whether abortion reforms have either a simultaneous or a one, two, three, four or five-year lagged association with fertility changes*.* The first six models consider the association without imputation of missing values in *abortion policy index 1*, while the second set of models includes imputed missing values in the independent variable. In combination, Table 1 shows that—using TWFE and without substantive control variables—*abortion policy index 1* does not have a significant association with the outcome in any of the models. Contemporaneous changes or changes with a 1, 2, 3, 4 or 5-year lag in *abortion policy index 1* are not significantly related to changes in the fertility rate. This is both the case when imputed missing values in independent variables are used (models 7–12) and when they are not (models 1–6). Controlling for country-constant characteristics and global trends, abortion liberalisation does not therefore have bivariate associations with fertility declines.

**Table 1:** FE models predicting the fertility rate, 1970–2019

	Model 1	Model 2	Model 3	Model 4	Model 5	Model 6
	Without imputation					
	No lag	1-year lag	2-year lag	3-year lag	4-year lag	5-year lag
Abortion policy index 1	0.018	0.020	0.026	0.030	0.034	0.034
	(0.037)	(0.038)	(0.038)	(0.038)	(0.038)	(0.038)
Country FE	Yes	Yes	Yes	Yes	Yes	Yes
Year FE	Yes	Yes	Yes	Yes	Yes	Yes
Country-specific linear time trend	No	No	No	No	No	No
Constant	5.202***	5.158***	5.019***	4.953***	4.880***	4.880***
	(0.110)	(0.110)	(0.110)	(0.109)	(0.107)	(0.107)
*N*	7831	7650	7288	7107	6926	6926
Countries	182	182	182	182	182	182

	Model 7	Model 8	Model 9	Model 10	Model 11	Model 12
	With imputation					
	No lag	1-year lag	2-year lag	3-year lag	4-year lag	5-year lag
Abortion policy index 1	0.022	0.022	0.023	0.024	0.025	0.026
	(0.034)	(0.034)	(0.034)	(0.034)	(0.033)	(0.033)
Country FE	Yes	Yes	Yes	Yes	Yes	Yes
Year FE	Yes	Yes	Yes	Yes	Yes	Yes
Country-specific linear time trend	No	No	No	No	No	No
Constant	5.238***	5.239***	5.237***	5.237***	5.237***	5.237***
	(0.102)	(0.102)	(0.101)	(0.099)	(0.099)	(0.098)
*N*	8175	8175	8175	8175	8175	8175
Countries	185	185	185	185	185	185

Standard errors in parentheses; *p < .05; **p < .01; ***p < .001. Robust standard errors are clustered at the country level

The variables for abortion reforms might not be significant in Table 1 because they may capture longitudinal changes of confounding factors. To assess this, Table 2 includes four pairs of models. All of them control for the 10 socio-economic and political factors discussed above. Models 1 and 5 use TWFE and capture within-country variations independently from global trends. Models 2 and 6 include country-specific linear time variables. They thus capture within-country variations independently from country-specific trends. Models 3 and 7 add year FE and country-specific linear time variables, reflecting only between-country variations net of general and country-specific trends. Models 4 and 8 only include year FE and hence only reflect cross-country variations.

**Table 2:** FE models predicting the fertility rate using *abortion policy index 1*, 1970–2019

	Model 1	Model 2	Model 3	Model 4	Model 5	Model 6	Model 7	Model 8
Multiple imputation	No	No	No	No	Yes	Yes	Yes	Yes
Abortion policy index 1	0.046*	− 0.002	− 0.007	0.046*	0.026	− 0.020	− 0.027	0.027
	(0.021)	(0.016)	(0.015)	(0.021)	(0.025)	(0.014)	(0.014)	(0.024)
Control variables								
Mean abortion policy index 1 in neighbouring countries	0.057	− 0.026	− 0.044	0.039	0.061	− 0.015	− 0.036	0.057
	(0.053)	(0.031)	(0.030)	(0.046)	(0.051)	(0.025)	(0.026)	(0.046)
GDP per capita logged	0.448**	0.131	0.084	0.452***	0.354***	0.059	0.053	0.384***
	(0.144)	(0.097)	(0.099)	(0.121)	(0.102)	(0.066)	(0.057)	(0.089)
Ratio female/male labour force participation	0.003	− 0.003	− 0.002	0.003	0.000	− 0.002	− 0.001	0.000
	(0.003)	(0.002)	(0.002)	(0.002)	(0.003)	(0.001)	(0.001)	(0.002)
Infant mortality logged	− 0.087	− 0.135	− 0.127	− 0.074	0.047	0.019	0.026	0.057
	(0.135)	(0.104)	(0.102)	(0.126)	(0.126)	(0.092)	(0.091)	(0.121)
Per cent urban population	− 0.016*	− 0.006	− 0.007	− 0.016**	− 0.015**	0.006	0.004	− 0.013**
	(0.007)	(0.010)	(0.009)	(0.006)	(0.006)	(0.008)	(0.008)	(0.004)
Median age of the population	0.014	0.045*	0.030	0.004	− 0.012	0.028	0.012	− 0.017
	(0.017)	(0.022)	(0.023)	(0.015)	(0.015)	(0.016)	(0.018)	(0.013)
Physicians per capita logged	− 0.218***	0.024	0.010	− 0.215***	− 0.200*	0.036	0.013	− 0.180*
	(0.060)	(0.040)	(0.040)	(0.056)	(0.077)	(0.039)	(0.038)	(0.074)
Average years of women’s education	− 0.527***	− 0.592***	− 0.620***	− 0.448***	− 0.485***	− 0.347***	− 0.349***	− 0.404***
	(0.077)	(0.096)	(0.103)	(0.054)	(0.067)	(0.057)	(0.060)	(0.050)
Contraceptive use	− 0.034***	− 0.039***	− 0.036***	− 0.034***	− 0.032***	− 0.038***	− 0.035***	− 0.032***
	(0.004)	(0.005)	(0.005)	(0.004)	(0.004)	(0.004)	(0.004)	(0.004)
Women civil liberty index	− 0.211	− 0.427**	− 0.260	− 0.225	− 0.164	− 0.505***	− 0.338*	− 0.145
	(0.255)	(0.154)	(0.170)	(0.238)	(0.268)	(0.133)	(0.144)	(0.253)
Country FE	Yes	Yes	Yes	No	Yes	Yes	Yes	No
Year FE	Yes	No	Yes	Yes	Yes	No	Yes	Yes
Country-specific linear time trend	No	Yes	Yes	No	No	Yes	Yes	No
Constant	5.952***	− 22.486	169.698***	5.694***	6.372***	13.592	20.599*	5.687***
	(1.490)	(19.311)	(25.637)	(1.248)	(1.099)	(12.829)	(10.216)	(0.956)
*N*	5329	5329	5329	5329	8175	8175	8175	8175
Countries	147	147	147	147	185	185	185	185

Standard errors in parentheses; *p < .05; **p < .01; ***p < .001. Robust standard errors are clustered at the country level

Regarding the control variables, two of them have a strong and robust association with the outcome: *average years of women’s education* and *contraceptive use*. In line with previous research (Martin, 1995), countries undergoing larger increases in women’s average years of education and contraceptive use display significantly lower increases in the fertility rate. Three other factors are related to the outcome in at least half of the models. Without using country trends (models 1 and 5) and focusing on cross-sectional differences (models 4 and 8), fertility levels are positively related to *GDP per capita logged* and negatively related to *physicians per capita* and the level of urbanisation. Moreover, the association with the other five socio-economic and political factors proves insufficiently robust: all else being equal, neither the changes or levels in the abortion policies of neighbouring countries, nor the ratio of female-to-male labour force participation, infant mortality rates, the median age, or more women’s civil liberties prove to be significantly and consistently associated with changes or levels in fertility levels.

More importantly, controlling for all these factors, *abortion policy index 1* is only significant in two models (in both cases with a positive association). In the TWFE model, changes in *abortion policy index 1* are positively related to increases in fertility rates (model 1). Moreover, countries with more liberal abortion policies display higher fertility rates (model 4). However, these two patterns prove to be linked to the range of considered countries, as by using multiple imputation (models 5 and 8) those effects become non-significant. The positive effect could thus be restricted to the set of countries included. Also, controlling for country-specific time-trends, *abortion policy index 1* proves to be unrelated to the outcome. Finally, the significance of the *abortion policy index 1* variable in models 1 and 4 is highly sensitive to the exclusion of single independent variables, such as GDP per capita (not shown), and to the inclusion of additional independent variables (e.g. Tables A7, A10).

The lack of a significant association between changes in abortion policy indexes and fertility levels could be influenced by the use of an aggregate index summing the legal grounds for having an induced abortion. Specific types of abortion liberalisation may shape fertility changes. A potentially highly relevant reform in this regard is the legalisation of abortion on request, which imposes the least restrictive conditions for terminating a pregnancy. To assess this, Table 3 replicates models 1 and 5 in Table 2—this time using the dichotomous variable *abortion on request*. Again, the models display four combinations of situations with/without country trends and with/without multiple imputation. Interestingly, abortion on request is negatively significantly related to the outcomes using multiple imputation. Legalising abortion on request is associated with an approximately 0.215 reduction in the TFR. However, this effect becomes non-significant once we consider countries without imputed data (models 1 and 2). Hence this association hinges substantially on the range of considered countries and the presence or not of controls for variables addressing the per cent adherents to different religions (Tables A7, A8).

**Table 3:** FE models predicting the fertility rate using *abortion on request*, 1970–2019

	Model 1	Model 2	Model 3	Model 4
	Without imputation		With multiple imputation	
Abortion on request	0.151	− 0.095	− 0.204**	− 0.215***
	(0.095)	(0.062)	(0.063)	(0.061)
Control variables				
Per cent neighbouring countries with abortion on request	0.878***	0.066	0.096	0.103
	(0.257)	(0.190)	(0.145)	(0.148)
GDP per capita logged	0.433**	0.075	0.054	0.043
	(0.135)	(0.097)	(0.065)	(0.056)
Ratio female/male labour force participation	0.003	− 0.002	− 0.002	− 0.001
	(0.003)	(0.002)	(0.001)	(0.001)
Infant mortality logged	− 0.118	− 0.123	0.020	0.031
	(0.127)	(0.102)	(0.092)	(0.089)
Per cent urban population	− 0.014*	− 0.006	0.005	0.004
	(0.007)	(0.009)	(0.008)	(0.008)
Median age of the population	0.002	0.031	0.028	0.009
	(0.016)	(0.023)	(0.016)	(0.017)
Physicians per capita logged	− 0.205***	0.012	0.037	0.016
	(0.058)	(0.039)	(0.038)	(0.037)
Average years of women’s education	− 0.514***	− 0.606***	− 0.345***	− 0.344***
	(0.075)	(0.107)	(0.056)	(0.059)
Contraceptive use	− 0.036***	− 0.037***	− 0.039***	− 0.037***
	(0.005)	(0.005)	(0.004)	(0.004)
Women civil liberty index	− 0.196	− 0.240	− 0.474***	− 0.310*
	(0.257)	(0.167)	(0.134)	(0.144)
Country FE	Yes	Yes	Yes	Yes
Year FE	Yes	Yes	Yes	Yes
Country-specific linear time trend	No	Yes	No	Yes
Constant	6.350***	− 25.797	13.268	6.353
	(1.389)	(20.747)	(12.667)	(13.161)
*N*	5329	5329	8175	8175
Countries	147	147	185	185

Standard errors in parentheses; *p < .05; **p < .01; ***p < .001. Robust standard errors are clustered at the country level

Thus far, we have assessed the demographic role of abortion decriminalisation based on the abortion policy dataset constructed by the authors. However, the results may prove to be sensitive to the categorisation of a few reforms. As noted above, two other cross-national, time-series datasets of abortion policy are available. This allows us to replicate models 1, 3, 5 and 7 in Table 2 using the two alternative abortion decriminalisation indexes. Table 4 displays these models with the association of abortion policy index 2 constructed by Bloom et al (2009), which covers the period 1970–2011. Using this alternative source, the results indicate some similarities and differences with respect to those in Table 2. The *average years of women’s education* and *contraceptive use* also have a negative and significant association with the outcome. *GDP per capita logged, women’s civil liberties index* and *the female/male labour force participation ratio* are significant in two models. Regarding the key variable of interest, *abortion policy index 2* is negative and significant in models 2 and 4. Using country trends, abortion reforms therefore appear to be significantly linked to fertility changes. However, in our preferred specifications without country trends (models 1 and 3)—which are less prone to being influenced by multicollinearity—, *abortion policy index 2* is positive and non-significant. Using this alternative indicator, the results do not therefore indicate a robust significant association between abortion reforms and fertility changes.

**Table 4:** FE models predicting the fertility rate using *abortion policy index 2*, 1970–2006

	Model 1	Model 2	Model 3	Model 4
	Without imputation		With multiple imputation	
Abortion policy index 2	0.033	− 0.025*	0.004	− 0.036**
	(0.018)	(0.011)	(0.022)	(0.011)
Control variables				
Mean abortion policy index 2 in neighbouring countries	0.010	0.007	0.046	− 0.006
	(0.038)	(0.023)	(0.038)	(0.018)
GDP per capita logged	0.530***	0.074	0.348***	0.032
	(0.121)	(0.107)	(0.100)	(0.065)
Ratio female/male labour force participation	0.001	− 0.005**	− 0.000	− 0.003*
	(0.003)	(0.002)	(0.002)	(0.001)
Infant mortality logged	− 0.159	− 0.118	0.072	0.031
	(0.148)	(0.110)	(0.138)	(0.093)
Per cent urban population	− 0.012	− 0.010	− 0.012	0.009
	(0.009)	(0.010)	(0.006)	(0.008)
Median age of the population	− 0.003	− 0.024	− 0.015	− 0.022
	(0.024)	(0.035)	(0.018)	(0.022)
Physicians per capita logged	− 0.205**	0.028	− 0.157	0.043
	(0.069)	(0.037)	(0.083)	(0.043)
Average years of women’s education	− 0.661***	− 0.544***	− 0.552***	− 0.272***
	(0.096)	(0.127)	(0.072)	(0.059)
Contraceptive use	− 0.036***	− 0.041***	− 0.033***	− 0.034***
	(0.006)	(0.006)	(0.005)	(0.004)
Women civil liberty index	− 0.005	− 0.344*	− 0.150	− 0.395**
	(0.210)	(0.154)	(0.256)	(0.119)
Country FE	Yes	Yes	Yes	Yes
Year FE	Yes	Yes	Yes	Yes
Country-specific linear time trend	No	Yes	No	Yes
Constant	6.387***	− 35.689	6.499***	15.581
	(1.389)	(22.892)	(1.156)	(13.664)
*N*	3754	3754	6719	6719
Countries	140	140	185	185

Standard errors in parentheses; *p < .05; **p < .01; ***p < .001. Robust standard errors are clustered at the country level

Table 5 replicates Table 2 using the third indicator: *abortion policy index 3*. This index was constructed by Forman-Rabinovici and Sommer (2018) and covers a shorter time span—1992–2015—than the previous two indexes. Probably because this shorter time span means that it captures fewer abortion reforms, this indicator does not prove to be significantly related to changes in the outcome. Although *abortion policy index 3* is negative in models 1–4, it is not significant in any of the four models in Table 5. Beyond the key variables of interest, in Table 5 the two control variables consistently related to changes in fertility noted above—*average years of women’s education* and *contraceptive use—*are still strongly related to the outcome.

**Table 5:** FE models predicting the fertility rate using *abortion policy index 3*, 1992–2015

	Model 1	Model 2	Model 3	Model 4
	Without imputation		With multiple imputation	
Abortion policy index 3	− 0.170	− 0.082	− 0.155	− 0.083
	(0.111)	(0.063)	(0.122)	(0.060)
Control variables				
Mean abortion policy index 3 in neighbouring countries	0.048	− 0.108	0.060	− 0.254*
	(0.222)	(0.116)	(0.220)	(0.124)
GDP per capita logged	0.133	0.097	0.207**	0.098
	(0.135)	(0.073)	(0.074)	(0.057)
Ratio female/male labour force participation	0.002	− 0.000	0.002	0.000
	(0.003)	(0.001)	(0.002)	(0.001)
Infant mortality logged	− 0.014	− 0.113	− 0.139	− 0.038
	(0.131)	(0.079)	(0.114)	(0.066)
Per cent urban population	− 0.023**	− 0.001	− 0.021**	0.012
	(0.008)	(0.012)	(0.007)	(0.013)
Median age of the population	0.036*	0.016	0.013	0.038
	(0.016)	(0.022)	(0.016)	(0.028)
Physicians per capita logged	− 0.078	− 0.004	− 0.109*	− 0.014
	(0.050)	(0.024)	(0.048)	(0.023)
Average years of women’s education	− 0.415***	− 0.351**	− 0.234**	− 0.059*
	(0.102)	(0.115)	(0.071)	(0.026)
Contraceptive use	− 0.027***	− 0.028***	− 0.023***	− 0.016***
	(0.005)	(0.005)	(0.004)	(0.004)
Women civil liberty index	− 0.725**	− 0.017	− 0.546*	− 0.029
	(0.270)	(0.140)	(0.210)	(0.087)
Country FE	Yes	Yes	Yes	Yes
Year FE	Yes	Yes	Yes	Yes
Country-specific linear time trend	No	Yes	No	Yes
Constant	7.507***	22.476	6.470***	97.642***
	(1.514)	(23.969)	(0.967)	(15.501)
*N*	3182	3182	4295	4295
Countries	144	144	182	182

Standard errors in parentheses; *p < .05; **p < .01; ***p < .001. Robust standard errors are clustered at the country level

### Sensitivity Analyses and Robustness Checks

We estimate a series of robustness checks and sensitivity analyses. First, Tables 2, 3, 4 and 5 address the instantaneous association of abortion policy reform with fertility changes. Second, to assess the potential delayed impact of reforms, Table A3 presents one-year-lag associations of the three policy indexes with and without imputed values. Table A4 also includes five-year-lag associations of the three policy indexes with and without imputed values. Third, the link between abortion policy and fertility rates may prove to be particularly robust for particular age groups of women. We therefore replicate the models in Table 2 but use the TFR of women in nine age groups (10–14, 15–19, 20–24, 25–29, 30–34, 35–39, 40–44, 45–49, 50–54) (Table A5).

Fourth, the TFR is a period-sensitive measure that is influenced by tempo effects. For example, women’s postponement of childbearing, which takes place in the studied setting, depresses the period-sensitive TFR, even though the number of children that women have over their life course does not change. In Table A6 we thus replicate the results using tempo-adjusted fertility rates, derived from the Human Fertility Database, as a dependent variable. This measure, proposed by Bongaarts and Feeney (1998), aims to remove tempo effects by using fertility data specified by birth order and age of the mother.^3^ Fifth, the introduction of controls for the percentage of adherents to key world religions (Tables A7, A8) could change the parameter estimate of abortion legislation. Sixth, we replicate the main model with a fully-balanced dataset including the whole period (Table A9). Seventh, we introduce an additional control of general government health expenditure (available for 2000–2019) (Table A10). Finally, we estimate the abortion reform-fertility link using difference-in-difference models (Figure A2).^4^

The evidence of these additional models proves to be revealing as it points to very limited significant associations between changes in abortion policy and changes in the TFR. Using 1-year and 5-year lagged variables, changes in two abortion policy indexes prove to be positively significantly related to the outcome. However, when using imputed values these associations become non-significant, suggesting that the non-imputed effects depends on the sample size (Tables A3, A4). Something similar occurs when using the TFR of different age groups. Without imputation, changes in *abortion policy index 1* are significantly related to TFR for (only) two of the nine age groups (in line with Myers (2017)), but when imputation is used all nine coefficients become non-significant (Table A5). When using adjusted fertility rates (Table A6), controlling for the percentage of adherents to major religions (Tables A7, A8), using a balanced dataset (Table A9) and controlling for public health expenditure (Table A10), *abortion policy index 1* is not significantly related to the outcome either. The three indicators of abortion policy also prove to be unrelated to shifts in the percentage of childless women (Table A13).

Table 3 provides indications that one form of abortion liberalisation—legalising abortion on request—may turn out to be more consistently related to changes in the outcome. To further explore the robustness of this on-request liberalisation-fertility link, we replicated Table A10 with controls for the percentage of adherents to different religions (Table A14) and estimated difference-in-difference models (Figure A2). In both instances, it becomes clear that the legalisation of abortion on request is not significantly related to the outcome.

## Conclusions

Seeking to shed light on the relationship between abortion decriminalisation and fertility changes, this study presents evidence of analyses that combine (a) three different indicators of abortion policy liberalism built by different research teams; (b) different time lags for abortion policy reforms; (c) the inclusion and exclusion of socio-economic control variables; and (d) with and without multiple imputation of independent variables. In a large majority of these models, abortion reforms prove to be non-significant predictors of the outcome. The evidence therefore indicates that both dimensions lack a sufficiently robust association.

The analysis conducted above is certainly not devoid of limitations. Most importantly, available data only allows us to explore the influence of abortion ‘law in the books’ rather than abortion ‘law in action’. This study measures abortion policy liberalism through the number of conditions under which national legislation allows induced abortion to be utilised (Bloom et al., 2009; Fernández, 2021; Forman-Rabinovici & Sommer, 2018). An alternative approach could have involved the use of indicators of the average national level of *access* to legal abortion. However, none of the available cross-national time-series provide estimates of effective abortion access. This is potentially relevant as several countries may display legal decoupling with liberal formal legislation on abortion and limited legal access to this health care procedure.

Another limitation is that the analysis discussed above did not include the handful of countries that legislate on abortion at subnational level and the reform-fertility link may therefore differ in those cases. Moreover, despite our best efforts to minimise the influence of unobserved heterogeneity (through the inclusion of multiple predictors of fertility changes, country-specific trends and FE), as in other global analyses, parameter estimates may still capture the influence of unconsidered factors. In addition, the analysis conducted above was globally oriented. The reform-fertility link may differ across world regions or historical periods.

Despite these limitations, the results of this study address an important and still unsolved debate on the role of abortion reforms in the fertility transition. The findings reported above are largely consistent with recent research reporting small differences in the incidence of abortion for countries with liberal and restrictive abortion legislation (Sedgh et al., 2016). However, they do challenge some previous work on the abortion reform-fertility link that assumes that abortion liberalisation fosters fertility decline (Bloom et al., 2009). One possible cause of this conundrum is that the relationship between changes in fertility and changes in abortion law may only be felt under certain circumstances. For instance, abortion liberalisation may only be demographically consequential if it is accompanied by other policy measures such as improvement in abortion access (González et al., 2018) and widespread knowledge of the reform (Assifi et al., 2016). Further research could therefore explore the scope conditions that facilitate a substantial relationship of abortion reform with demographic changes. The inconsistency between the main conclusion of this study and the conclusions from Bloom et al. (2009) may also be due to the fact that this latter study included fewer control variables and a shorter time period.

Until more cross-national time-series research is conducted on the matter, this article reports a largely null association between an average abortion reform and changes in fertility. This finding has two important policy implications. First, it is directly relevant to the emerging interest in harnessing a demographic dividend. International organisation and state actors concur that low-income countries with high fertility can accelerate economic growth by reducing fertility levels that will, in turn, affect economic resources from raising children to skills formation and capital accumulation (Bloom et al., 2009; Eloundou-Enyegue, 2013). Our study suggests that abortion reform is not a promising strategy to harness such a dividend, as it has not been associated with fertility decline in the past. Second, the results are also relevant to countries in the opposite situation: very low fertility rates and low abortion liberalism (Reuters, 2017). The domestic concerns raised in this latter group of countries about the potential accelerating influence of abortion decriminalisation on population ageing would be unjustified since, on average, worldwide abortion reform is generally unrelated to fertility changes.

### Supplementary Information

Below is the link to the electronic supplementary material.

Supplementary file1 (DOCX 212 kb)

## Footnotes

1 A given value in an additive index of abortion liberalism could potentially include different combinations of grounds. However, that is not the case in *abortion policy index 1*. Country-years with a value 2 generally allow abortion under life and health risks (100% and 90%, respectively), but very rarely in cases of rape, foetal impairment and for socio-economic reasons (8%, 2% and 0%, respectively). Country-years with a value 4 generally allow abortion under conditions of life risks, health risks, rape and foetal impairment (100%, 100%, 81%, 93%, respectively), but rarely under conditions of socio-economic grounds (26%). This evidence indicates very limited heterogeneity in the grounds for which abortion is legal at values 2 and 4.

2 Imputed values were estimated using the Amelia II program (Honaker et al., 2013). The imputation model included country-specific intercepts and second-order polynomials (and a ridge prior of 1%) to capture country-specific trends. To ease convergence, the estimation model only included variables without lags. Lagged variables were constructed based on previously imputed values.

3 In Tables A5, A6 and A9 we only utilise *abortion policy index 1* because it has the longest time span of the three indicators and in our view has the highest quality of the three.

4 Of the control variables added in the Online Appendix tables, only *government health expenditure* displays consistently significant coefficients. Increases in *government health expenditure* have, ceteris paribus, a positive effect on TFR changes.
