# Supplementary material for: Does Abortion Liberalisation Accelerate Fertility Decline? A Worldwide Time-Series Analysis
Source: Eur J Popul. 2023 Dec 5;39(1):36. doi: 10.1007/s10680-023-09687-y (PMC10697910; doi:10.1007/s10680-023-09687-y)
Supplement: Supplementary file 1 — Supplementary file1 (DOCX 212 kb) [file 10680_2023_9687_MOESM1_ESM.docx]

**Online Appendix of the manuscript entitled “Does Abortion Liberalization Accelerate Fertility Decline? A Worldwide Time-series Analysis”**

| Table A1. Descriptive statistics of all variables without multiple imputation and centering | | | | | |
| --- | --- | --- | --- | --- | --- |
|  | N | Mean | SD | Min | Max |
| Total fertility rate | 8,175 | 3.795 | 1.955 | 0.914 | 8.864 |
| Abortion policy index 1 | 7,831 | 2.715 | 2.142 | 0.000 | 6.000 |
| Abortion policy index 2 | 5,431 | 3.307 | 2.380 | 0.000 | 7.000 |
| Abortion policy index 3 | 4,241 | 0.414 | 0.396 | 0.000 | 1.000 |
| Abortion on request | 7,831 | 0.197 | 0.398 | 0.000 | 1.000 |
| Mean value of abortion policy index 1 in neighboring countries | 7,997 | 2.886 | 1.642 | 0.000 | 6.000 |
| Mean value of abortion policy index 2 in neighboring countries | 5,657 | 3.474 | 1.802 | 0.000 | 7.000 |
| Mean value of abortion policy index 3 in neighboring countries | 4,226 | 0.446 | 0.310 | 0.007 | 1.000 |
| Percent countries that have liberalized abortion on request | 7,997 | 0.214 | 0.320 | 0.000 | 1.000 |
| GDP per capita log | 7,282 | 8.236 | 1.438 | 5.040 | 12.161 |
| Ratio female/male labor force participation | 6,945 | 61.616 | 23.343 | 2.973 | 124.018 |
| Infant mortality log | 8,175 | 3.362 | 1.089 | 0.139 | 5.581 |
| Urban population | 8,175 | 50.638 | 24.174 | 2.845 | 100.000 |
| Median age of the population | 8,175 | 23.484 | 8.002 | 13.113 | 54.282 |
| Physicians per capita log | 7,495 | 6.797 | 3.254 | 0.020 | 13.304 |
| Av. years of women’s education | 7,628 | 33.422 | 22.496 | 0.000 | 79.300 |
| Contraception use | 7,795 | 3.932 | 1.537 | -0.916 | 6.736 |
| Women civil liberties | 7,496 | 0.605 | 0.272 | 0.000 | 0.981 |
| TFR 10-14 | 8,175 | 4.152 | 5.109 | 0.000 | 44.044 |
| TFR 15-19 | 8,175 | 74.394 | 53.111 | 0.876 | 249.244 |
| TFR 20-24 | 8,175 | 171.062 | 79.379 | 7.180 | 379.075 |
| TFR 25-29 | 8,175 | 185.476 | 75.196 | 35.645 | 382.013 |
| TFR 30-34 | 8,175 | 153.277 | 74.191 | 22.112 | 357.717 |
| TFR 35-39 | 8,175 | 104.208 | 68.499 | 7.199 | 297.480 |
| TFR 40-44 | 8,175 | 50.659 | 44.999 | 1.416 | 211.939 |
| TFR 45-49 | 8,175 | 15.331 | 18.230 | 0.000 | 104.601 |
| TFR 50-54 | 8,175 | 1.114 | 1.914 | 0.000 | 22.721 |
| Adjusted TFR | 840 | 1.832 | 0.275 | 1.191 | 3.169 |
| Perc. childless | 178 | 12.029 | 4.521 | 1.300 | 27.000 |
| Agricultural labor force | 4,809 | 31.101 | 23.933 | 0.403 | 92.592 |
| Health expenditure | 3,514 | 3.072 | 2.164 | 0.062 | 22.254 |
| Percent Catholics | 6,337 | 3.027 | .344 | 0 | .980 |
| Percent Muslims | 6,337 | .254 | .367 | 0 | 1 |
|  |  |  |  |  |  |

| Table A2. Correlation Matrix of All Variables | | | | | | | | | | | | | | | | | | |
| --- | --- | --- | --- | --- | --- | --- | --- | --- | --- | --- | --- | --- | --- | --- | --- | --- | --- | --- |
|  | Total fertility rate | Abortion policy index 1 | Abortion policy index 3 | Abortion policy index 2 | Abortion on request | Mean value in abortion policy index 1 in neigh. | Mean value in abortion policy index 2 in neigh. | Mean value in abortion policy index 3 in neigh. | Percent countries that have liberalized abortion on request | GDP per capita log | Ratio female/male labor force participation | Infant mortality | Urban population | Median age of the population | Physicians per capita | Av. years of women’s educ. | Contraception use |  |
| Abortion policy index 1 | -0.32 | 1.00 |  |  |  |  |  |  |  |  |  |  |  |  |  |  |  |  |
| Abortion policy index 3 | -0.42 | 0.93 | 1.00 |  |  |  |  |  |  |  |  |  |  |  |  |  |  |  |
| Abortion policy index 2 | -0.32 | 0.94 | 0.88 | 1.00 |  |  |  |  |  |  |  |  |  |  |  |  |  |  |
| Abortion on request | -0.12 | 0.78 | 0.62 | 0.82 | 1.00 |  |  |  |  |  |  |  |  |  |  |  |  |  |
| Mean value of abortion policy index 1 in neig. | -0.11 | 0.79 | 0.73 | 0.78 | 0.66 | 1.00 |  |  |  |  |  |  |  |  |  |  |  |  |
| Mean value of abortion policy index 2 in neighboring countries | -0.18 | 0.77 | 0.77 | 0.75 | 0.58 | 0.96 | 1.00 |  |  |  |  |  |  |  |  |  |  |  |
| Mean value of abortion policy index 3 in neighboring countries | -0.18 | 0.79 | 0.76 | 0.75 | 0.61 | 0.97 | 0.98 | 1.00 |  |  |  |  |  |  |  |  |  |  |
| Percent countries that have liberalized abortion on request | -0.11 | 0.74 | 0.69 | 0.71 | 0.59 | 0.95 | 0.92 | 0.94 | 1.00 |  |  |  |  |  |  |  |  |  |
| GDP per capita log | 0.37 | 0.02 | 0.09 | 0.02 | -0.12 | -0.07 | -0.05 | -0.07 | 0.01 | 1.00 |  |  |  |  |  |  |  |  |
| Ratio female/male labor force participation | 0.01 | 0.70 | 0.61 | 0.65 | 0.49 | 0.70 | 0.67 | 0.68 | 0.54 | -0.10 | 1.00 |  |  |  |  |  |  |  |
| Infant mortality | -0.11 | -0.03 | -0.14 | -0.03 | 0.18 | 0.11 | 0.07 | 0.07 | 0.03 | -0.86 | 0.03 | 1.00 |  |  |  |  |  |  |
| Urban population | 0.48 | -0.25 | -0.18 | -0.19 | -0.17 | -0.12 | -0.07 | -0.09 | -0.09 | 0.36 | -0.09 | -0.33 | 1.00 |  |  |  |  |  |
| Median age of the population | -0.34 | 0.57 | 0.66 | 0.47 | 0.21 | 0.31 | 0.36 | 0.41 | 0.34 | 0.30 | 0.39 | -0.48 | -0.01 | 1.00 |  |  |  |  |
| Physicians per capita | -0.42 | 0.51 | 0.45 | 0.46 | 0.31 | 0.24 | 0.20 | 0.22 | 0.22 | -0.15 | 0.30 | 0.04 | -0.25 | 0.45 | 1.00 |  |  |  |
| Av. years of women’s educ. | 0.07 | 0.31 | 0.37 | 0.30 | 0.32 | 0.54 | 0.58 | 0.60 | 0.57 | 0.25 | 0.20 | -0.27 | 0.34 | 0.22 | -0.16 | 1.00 |  |  |
| Contraception use | 0.33 | 0.03 | 0.03 | 0.10 | -0.02 | -0.02 | 0.00 | -0.07 | 0.02 | 0.75 | 0.00 | -0.67 | 0.40 | 0.07 | -0.14 | 0.09 | 1.00 |  |
| Women civil liberties | 0.18 | 0.08 | 0.14 | 0.04 | -0.14 | 0.01 | 0.03 | 0.00 | 0.05 | 0.77 | -0.05 | -0.67 | 0.08 | 0.24 | -0.05 | 0.20 | 0.59 |  |


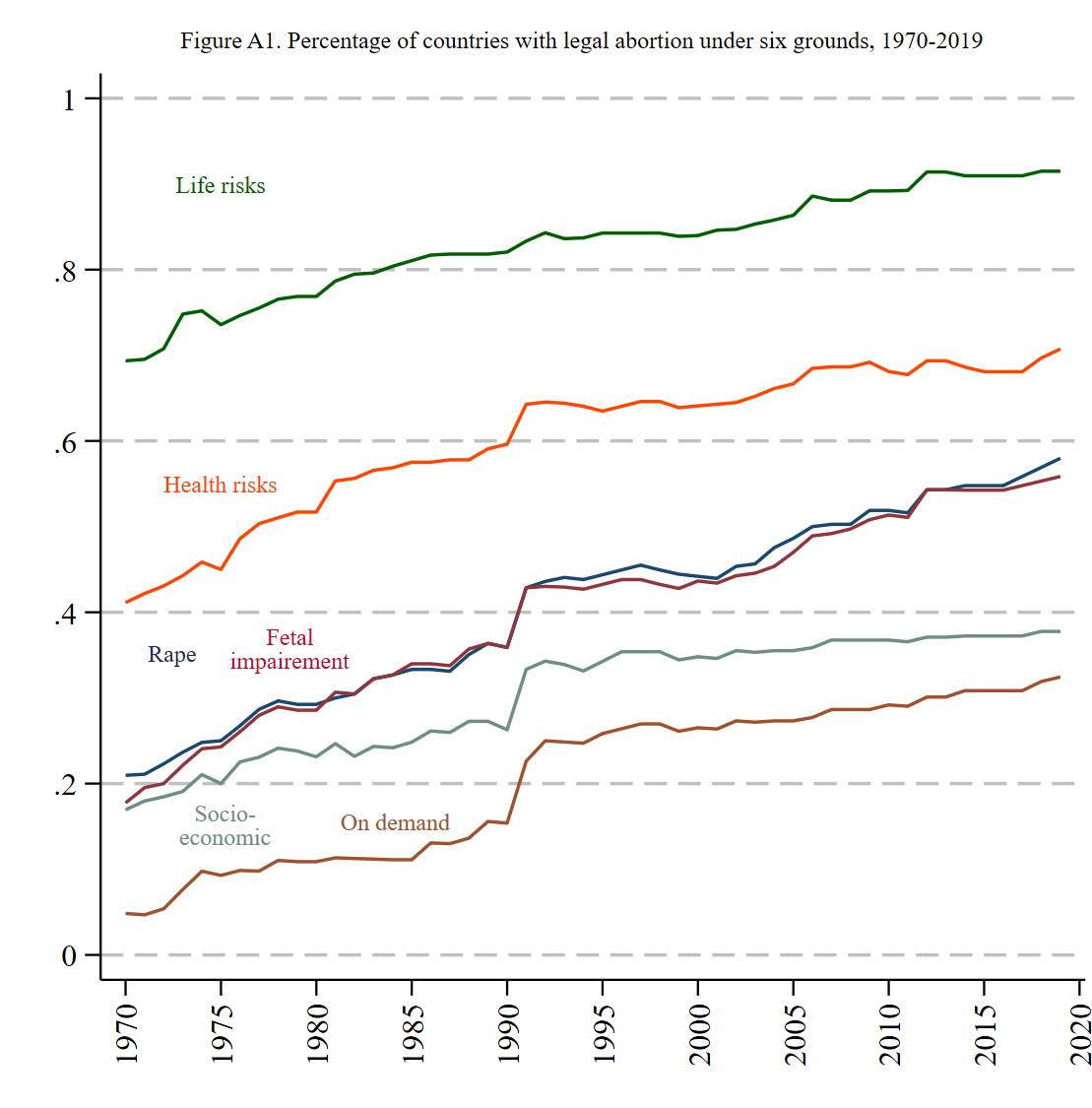


| Table A3. FE Models Predicting the Fertility Rate Using 1-year lag | | | | | | |
| --- | --- | --- | --- | --- | --- | --- |
|  | No imputation | Imputation | No imputation | Imputation | No imputation | Imputation |
|  | Model 1 | Model 2 | Model 3 | Model 4 | Model 5 | Model 6 |
| Abortion policy index 1_(t-1)_ | 0.048* | 0.028 |  |  |  |  |
|  | (0.021) | (0.025) |  |  |  |  |
| Abortion policy index 2_(t-1)_ |  |  | 0.036* | 0.008 |  |  |
|  |  |  | (0.018) | (0.023) |  |  |
| Abortion policy index 3_(t-1)_ |  |  |  |  | -0.175 | -0.139 |
|  |  |  |  |  | (0.108) | (0.123) |
| Control variables |  |  |  |  |  |  |
| Mean value in abortion policy index 1 in ne. countries_(t-1)_ | 0.051 | 0.057 |  |  |  |  |
|  | (0.052) | (0.050) |  |  |  |  |
| Mean value in abortion policy index 2 in ne. countries_(t-1)_ |  |  | 0.006 | 0.043 |  |  |
|  |  |  | (0.038) | (0.037) |  |  |
| Mean value in abortion policy index 3 in ne. countries_(t-1)_ |  |  |  |  | 0.046 | 0.084 |
|  |  |  |  |  | (0.220) | (0.222) |
| GDP per capita logged_(t-1)_ | 0.440** | 0.369*** | 0.515*** | 0.367*** | 0.148 | 0.212** |
|  | (0.139) | (0.101) | (0.119) | (0.099) | (0.131) | (0.069) |
| Ratio female/male labor force participation rate_(t-1)_ | 0.002 | 0.001 | 0.001 | 0.000 | 0.002 | 0.002 |
|  | (0.003) | (0.002) | (0.003) | (0.002) | (0.003) | (0.002) |
| Infant mortality logged_(t-1)_ | -0.057 | 0.054 | -0.109 | 0.095 | -0.001 | -0.132 |
|  | (0.138) | (0.127) | (0.149) | (0.139) | (0.132) | (0.115) |
| Percent urban population_(t-1)_ | -0.016* | -0.016** | -0.012 | -0.013* | -0.023** | -0.020** |
|  | (0.007) | (0.006) | (0.009) | (0.006) | (0.008) | (0.007) |
| Median age_(t-1)_ | 0.030 | 0.000 | 0.019 | -0.001 | 0.050** | 0.026 |
|  | (0.017) | (0.015) | (0.025) | (0.018) | (0.016) | (0.016) |
| Physicians per capita logged_(t-1)_ | -0.210*** | -0.209** | -0.203** | -0.163* | -0.075 | -0.114* |
|  | (0.060) | (0.077) | (0.070) | (0.082) | (0.050) | (0.051) |
| Average years of women’s education_(t-1)_ | -0.522*** | -0.481*** | -0.663*** | -0.545*** | -0.376*** | -0.229** |
|  | (0.077) | (0.066) | (0.098) | (0.070) | (0.100) | (0.070) |
| Contraceptive use_(t-1)_ | -0.033*** | -0.032*** | -0.034*** | -0.033*** | -0.027*** | -0.023*** |
|  | (0.005) | (0.004) | (0.006) | (0.005) | (0.005) | (0.004) |
| Women civil liberty index_(t-1)_ | -0.216 | -0.160 | -0.025 | -0.136 | -0.757** | -0.548** |
|  | (0.254) | (0.266) | (0.209) | (0.251) | (0.289) | (0.204) |
| Country FE | Yes | Yes | Yes | Yes | Yes | Yes |
| Year FE | Yes | Yes | Yes | Yes | Yes | Yes |
| Country-specific linear trends | No | No | No | No | No | No |
| Constant | 5.483*** | 5.806*** | 5.806*** | 5.973*** | 6.686*** | 6.004*** |
|  | (1.472) | (1.379) | (1.379) | (1.094) | (1.481) | (0.948) |
| N | 5,257 | 3,754 | 3,182 | 8,175 | 6,719 | 4,295 |
| Countries | 147 | 140 | 144 | 185 | 185 | 182 |

Note: Standard errors in parentheses; ^*^ *p* < .05, ^**^ *p* < .01, ^***^ *p* < .001. Robust standard errors are clustered at the country level.

| Table A4. FE Models Predicting the Fertility Rate Using 5-year lag | | | | | | |
| --- | --- | --- | --- | --- | --- | --- |
|  | No imputation | Imputation | No imputation | Imputation | No imputation | Imputation |
|  | Model 1 | Model 2 | Model 3 | Model 4 | Model 5 | Model 6 |
| Abortion policy index 1_(t-5)_ | 0.059** | 0.033 |  |  |  |  |
|  | (0.021) | (0.024) |  |  |  |  |
| Abortion policy index 2_(t-5)_ |  |  | 0.055** | 0.024 |  |  |
|  |  |  | (0.021) | (0.021) |  |  |
| Abortion policy index 3_(t-5)_ |  |  |  |  | -0.104 | -0.034 |
|  |  |  |  |  | (0.099) | (0.111) |
| Control variables |  |  |  |  |  |  |
| Mean value in abortion policy index 1 in ne. countries_(t-5)_ | 0.031 | 0.046 |  |  |  |  |
|  | (0.050) | (0.046) |  |  |  |  |
| Mean value in abortion policy index 2 in ne. countries_(t-5)_ |  |  | -0.006 | 0.032 |  |  |
|  |  |  | (0.040) | (0.037) |  |  |
| Mean value in abortion policy index 3 in ne. countries_(t-5)_ |  |  |  |  | 0.104 | 0.159 |
|  |  |  |  |  | (0.208) | (0.191) |
| GDP per capita logged_(t-5)_ | 0.370** | 0.350*** | 0.422*** | 0.355*** | 0.128 | 0.141* |
|  | (0.128) | (0.097) | (0.124) | (0.097) | (0.114) | (0.066) |
| Ratio female/male labor force participation rate_(t-5)_ | 0.003 | 0.002 | 0.003 | 0.002 | 0.002 | 0.000 |
|  | (0.003) | (0.002) | (0.003) | (0.002) | (0.002) | (0.002) |
| Infant mortality logged_(t-5)_ | 0.042 | 0.134 | 0.073 | 0.247 | 0.024 | -0.041 |
|  | (0.152) | (0.132) | (0.162) | (0.136) | (0.128) | (0.113) |
| Percent urban population_(t-5)_ | -0.016* | -0.017** | -0.014 | -0.017* | -0.021** | -0.019** |
|  | (0.008) | (0.006) | (0.009) | (0.007) | (0.008) | (0.007) |
| Median age_(t-5)_ | 0.089*** | 0.050** | 0.095*** | 0.052** | 0.096*** | 0.076*** |
|  | (0.019) | (0.016) | (0.026) | (0.019) | (0.017) | (0.017) |
| Physicians per capita logged_(t-5)_ | -0.181** | -0.240*** | -0.183** | -0.193* | -0.049 | -0.126* |
|  | (0.060) | (0.072) | (0.066) | (0.074) | (0.045) | (0.061) |
| Average years of women’s education_(t-5)_ | -0.492*** | -0.457*** | -0.608*** | -0.503*** | -0.256** | -0.192** |
|  | (0.083) | (0.060) | (0.104) | (0.063) | (0.091) | (0.066) |
| Contraceptive use_(t-5)_ | -0.027*** | -0.028*** | -0.027*** | -0.028*** | -0.022*** | -0.019*** |
|  | (0.005) | (0.004) | (0.006) | (0.005) | (0.004) | (0.004) |
| Women civil liberty index_(t-5)_ | -0.219 | -0.149 | -0.106 | -0.105 | -0.824* | -0.276 |
|  | (0.254) | (0.251) | (0.227) | (0.236) | (0.319) | (0.166) |
| Country FE | Yes | Yes | Yes | Yes | Yes | Yes |
| Year FE | Yes | Yes | Yes | Yes | Yes | Yes |
| Country-specific linear trends | No | No | No | No | No | No |
| Constant | 3.822* | 3.619* | 4.262** | 4.494*** | 3.935*** | 4.502*** |
|  | (1.480) | (1.454) | (1.408) | (1.103) | (1.113) | (0.876) |
| N | 4,787 | 3,754 | 3,053 | 8,175 | 6,719 | 4,295 |
| Countries | 145 | 140 | 142 | 185 | 185 | 182 |

Note: Standard errors in parentheses; ^*^ *p* < .05, ^**^ *p* < .01, ^***^ *p* < .001. Robust standard errors are clustered at the country level.

| Table A5. FE Models Predicting the Fertility Rate, 1970-2019 | | | | | | |  |  |  |
| --- | --- | --- | --- | --- | --- | --- | --- | --- | --- |
|  | Model 1 | Model 2 | Model 3 | Model 4 | Model 5 | Model 6 | Model 7 | Model 8 | Model 9 |
| Without imputation | | | | | | | | | |
|  | TFR 10-14 | TFR 15-19 | TFR 20-24 | TFR 25-29 | TFR 30-34 | TFR 35-39 | TFR 40-44 | TFR 45-49 | TFR 50-54 |
| Abortion policy index 1 | 0.104 | 0.684 | 0.677 | 1.253 | 2.044 | 1.938 | 1.561** | 0.712** | 0.043 |
|  | (0.078) | (0.606) | (0.743) | (1.036) | (1.342) | (1.037) | (0.546) | (0.237) | (0.038) |
| Country FE | Yes | Yes | Yes | Yes | Yes | Yes | Yes | Yes | Yes |
| Year FE | Yes | Yes | Yes | Yes | Yes | Yes | Yes | Yes | Yes |
| Country-specific linear time trend | No | No | No | No | No | No | No | No | No |
| Constant | 5.708 | 50.685 | 145.805* | 278.969*** | 307.215*** | 251.231*** | 116.338*** | 32.784 | -1.906 |
|  | (4.678) | (50.651) | (68.836) | (73.971) | (72.726) | (54.630) | (27.420) | (17.869) | (3.444) |
| N | 5,329 | 5,329 | 5,329 | 5,329 | 5,329 | 5,329 | 5,329 | 5,329 | 5,329 |
| Countries | 147 | 147 | 147 | 147 | 147 | 147 | 147 | 147 | 147 |
|  |  |  |  |  |  |  |  |  |  |
|  | Model 10 | Model 11 | Model 12 | Model 13 | Model 14 | Model 15 | Model 16 | Model 17 | Model 18 |
| With imputation | | | | | | | | | |
|  | TFR 10-14 | TFR 15-19 | TFR 20-24 | TFR 25-29 | TFR 30-34 | TFR 35-39 | TFR 40-44 | TFR 45-49 | TFR 50-54 |
| Abortion policy index 1 | 0.122 | 0.882 | 0.607 | 0.145 | 1.366 | 0.955 | 0.739 | 0.262 | -0.010 |
|  | (0.084) | (0.595) | (0.678) | (1.154) | (1.367) | (1.199) | (0.714) | (0.283) | (0.043) |
| Country FE | Yes | Yes | Yes | Yes | Yes | Yes | Yes | Yes | Yes |
| Year FE | Yes | Yes | Yes | Yes | Yes | Yes | Yes | Yes | Yes |
| Country-specific linear time trend | No | No | No | No | No | No | No | No | No |
| Constant | -1.376 | 43.067 | 204.594*** | 319.656*** | 322.226*** | 251.406*** | 110.334*** | 21.459 | -1.689 |
|  | (3.977) | (35.861) | (47.898) | (51.374) | (53.308) | (43.960) | (24.579) | (12.252) | (2.224) |
| N | 8,175 | 8,175 | 8,175 | 8,175 | 8,175 | 8,175 | 8,175 | 8,175 | 8,175 |
| Countries | 185 | 185 | 185 | 185 | 185 | 185 | 185 | 185 | 185 |

Notes: Standard errors in parentheses; ^*^ *p* < .05, ^**^ *p* < .01, ^***^ *p* < .001. Robust standard errors are clustered at the country level. Models estimated with controls for *mean index of abortion index 1 in neighboring countries, GDP per capita, ratio of female/male labor force participation rate, infant mortality logged, percent urban population, median age, physicians per capita logged, average years of women’s education, contraceptive use, women’s civil liberties index.*

| Table A6. FE Models Predicting the Adjusted Fertility Rate Using *Abortion Policy Index 1*, 1970-2019 | |  |
| --- | --- | --- |
|  | Model 1 |  |
|  | Without imputation | |
| Abortion policy index 1 | 0.011 |  |
|  | (0.015) |  |
| Control variables |  |  |
| Mean abortion policy index 1 in neighboring countries | -0.010 |  |
|  | (0.059) |  |
| GDP per capita logged | 0.199 |  |
|  | (0.145) |  |
| Ratio female/male labor force participation | 0.004 |  |
|  | (0.002) |  |
| Infant mortality logged | 0.158* |  |
|  | (0.062) |  |
| Percent urban population | -0.005 |  |
|  | (0.005) |  |
| Median age of the population | -0.014 |  |
|  | (0.015) |  |
| Physicians per capita logged | 0.038 |  |
|  | (0.170) |  |
| Average years of women’s education | -0.193** |  |
|  | (0.060) |  |
| Contraceptive use | -0.002 |  |
|  | (0.005) |  |
| Women civil liberty index | -0.591*** |  |
|  | (0.149) |  |
| Country FE | Yes |  |
| Year FE | Yes |  |
| Country-specific linear time trend | No |  |
| Constant | 2.699 |  |
|  | (1.513) |  |
| N | 707 |  |
| Countries | 24 |  |

Note: Standard errors in parentheses; ^*^ *p* < .05, ^**^ *p* < .01, ^***^ *p* < .001. Robust standard errors are clustered at the country level. Given the small number of countries with a fully-balanced dataset, in this test we restrict the analysis to the abortion policy index with the largest temporal and country coverage.

| Table A7. FE Models Predicting the Fertility Rate Using Controls for Religiosity, 1970-2010 | | | | |
| --- | --- | --- | --- | --- |
|  | Model 1 | Model 2 | Model 3 |  |
|  | Without imputation | Without imputation | Without imputation |  |
| Abortion policy index 1 | 0.035 |  |  |  |
|  | (0.019) |  |  |  |
| Abortion policy index 2 |  | 0.025 |  |  |
|  |  | (0.017) |  |  |
| Abortion policy index 3 |  |  | -0.129 |  |
|  |  |  | (0.117) |  |
| Control variables |  |  |  |  |
| Mean abortion policy index 1 in neighboring countries | 0.018 |  |  |  |
|  | (0.048) |  |  |  |
| Mean abortion policy index 2 in neighboring countries |  | 0.009 |  |  |
|  |  | (0.036) |  |  |
| Mean abortion policy index 3 in neighboring countries |  |  | 0.222 |  |
|  |  |  | (0.204) |  |
| GDP per capita logged | 0.532*** | 0.566*** | 0.065 |  |
|  | (0.119) | (0.117) | (0.113) |  |
| Ratio female/male labor force participation | 0.002 | 0.001 | 0.002 |  |
|  | (0.003) | (0.002) | (0.003) |  |
| Infant mortality logged | -0.062 | -0.057 | 0.005 |  |
|  | (0.151) | (0.151) | (0.121) |  |
| Percent urban population | -0.014 | -0.013 | -0.025** |  |
|  | (0.008) | (0.008) | (0.009) |  |
| Median age of the population | 0.003 | -0.007 | 0.044* |  |
|  | (0.022) | (0.024) | (0.020) |  |
| Physicians per capita logged | -0.206** | -0.198** | -0.045 |  |
|  | (0.066) | (0.068) | (0.050) |  |
| Average years of women’s education | -0.597*** | -0.621*** | -0.494*** |  |
|  | (0.088) | (0.094) | (0.108) |  |
| Contraceptive use | -0.034*** | -0.036*** | -0.026*** |  |
|  | (0.005) | (0.005) | (0.005) |  |
| Women civil liberty index | -0.159 | -0.077 | -0.499* |  |
|  | (0.225) | (0.200) | (0.234) |  |
| Percent Catholic | -1.223 | -1.798* | 0.065 |  |
|  | (0.720) | (0.757) | (0.435) |  |
| Percent Muslims | 2.008 | 1.681 | 1.319 |  |
|  | (1.240) | (1.168) | (0.855) |  |
| Country FE | Yes | Yes | Yes |  |
| Year FE | Yes | Yes | Yes |  |
| Country-specific linear time trend | No | No | No |  |
| Constant | 5.630*** | 5.856*** | 7.625*** |  |
|  | (1.388) | (1.353) | (1.403) |  |
| Countries | 4,191 | 3,729 | 2,471 |  |
| N | 143 | 140 | 141 |  |

Note: Standard errors in parentheses; ^*^ *p* < .05, ^**^ *p* < .01, ^***^ *p* < .001. Robust standard errors are clustered at the country level.

| Table A8. FE Models Predicting the Fertility Rate Using Controls for Religiosity, 1970-2010 | | | | |
| --- | --- | --- | --- | --- |
|  | Model 1 | Model 2 | Model 3 |  |
|  | With imputation | With imputation | With imputation |  |
| Abortion policy index 1 | 0.012 |  |  |  |
|  | (0.025) |  |  |  |
| Abortion policy index 2 |  | 0.003 |  |  |
|  |  | (0.022) |  |  |
| Abortion policy index 3 |  |  | -0.116 |  |
|  |  |  | (0.134) |  |
| Control variables |  |  |  |  |
| Mean abortion policy index 1 in neighboring countries | 0.027 |  |  |  |
|  | (0.049) |  |  |  |
| Mean abortion policy index 2 in neighboring countries |  | 0.040 |  |  |
|  |  | (0.038) |  |  |
| Mean abortion policy index 3 in neighboring countries |  |  | 0.191 |  |
|  |  |  | (0.229) |  |
| GDP per capita logged | 0.339*** | 0.341*** | 0.192** |  |
|  | (0.097) | (0.096) | (0.068) |  |
| Ratio female/male labor force participation | -0.000 | -0.000 | 0.001 |  |
|  | (0.002) | (0.002) | (0.002) |  |
| Infant mortality logged | 0.101 | 0.108 | -0.077 |  |
|  | (0.139) | (0.139) | (0.123) |  |
| Percent urban population | -0.014* | -0.013* | -0.023** |  |
|  | (0.006) | (0.006) | (0.008) |  |
| Median age of the population | -0.014 | -0.014 | 0.003 |  |
|  | (0.019) | (0.018) | (0.018) |  |
| Physicians per capita logged | -0.146 | -0.144 | -0.066 |  |
|  | (0.082) | (0.082) | (0.052) |  |
| Average years of women’s education | -0.532*** | -0.533*** | -0.245** |  |
|  | (0.071) | (0.071) | (0.076) |  |
| Contraceptive use | -0.033*** | -0.033*** | -0.020*** |  |
|  | (0.005) | (0.005) | (0.004) |  |
| Women civil liberty index | -0.147 | -0.158 | -0.393* |  |
|  | (0.257) | (0.255) | (0.180) |  |
| Percent Catholic | -1.166* | -1.139* | -0.036 |  |
|  | (0.574) | (0.567) | (0.345) |  |
| Percent Muslims | 2.009** | 2.053** | 0.838 |  |
|  | (0.706) | (0.733) | (0.520) |  |
| Country FE | Yes | Yes | Yes |  |
| Year FE | Yes | Yes | Yes |  |
| Country-specific linear time trend | No | No | No |  |
| Constant | 6.289*** | 6.201*** | 6.221*** |  |
|  | (1.123) | (1.124) | (0.953) |  |
| N | 6,537 | 6,537 | 3,385 |  |
| Countries | 184 | 184 | 181 |  |

Note: Standard errors in parentheses; ^*^ *p* < .05, ^**^ *p* < .01, ^***^ *p* < .001. Robust standard errors are clustered at the country level.

| Table A9. FE Models Predicting the Fertility Rate Using *Abortion Policy Index 1* and using a balanced dataset, 1970-2019 | |
| --- | --- |
|  | Model 1 |
|  | Without imputation |
| Abortion policy index 1 | 0.042 |
|  | (0.027) |
| Control variables |  |
| Mean abortion policy index 1 in neighboring countries | 0.187* |
|  | (0.075) |
| GDP per capita logged | 0.380 |
|  | (0.250) |
| Ratio female/male labor force participation | 0.006 |
|  | (0.004) |
| Infant mortality logged | -0.551** |
|  | (0.186) |
| Percent urban population | -0.021* |
|  | (0.010) |
| Median age of the population | 0.055 |
|  | (0.029) |
| Physicians per capita logged | -0.006 |
|  | (0.097) |
| Average years of women’s education | -0.658*** |
|  | (0.101) |
| Contraceptive use | -0.020** |
|  | (0.007) |
| Women civil liberty index | -0.352 |
|  | (0.280) |
| Country FE | Yes |
| Year FE | Yes |
| Country-specific linear time trend | No |
| Constant | 7.222** |
|  | (2.511) |
| N | 1,450 |
| Countries | 29 |

Note: Standard errors in parentheses; ^*^ *p* < .05, ^**^ *p* < .01, ^***^ *p* < .001. Robust standard errors are clustered at the country level. Given the small number of countries with a fully-balanced dataset, in this test we restrict the analysis to the abortion policy index with the largest temporal and country coverage.

| Table A10. FE Models Predicting the Fertility Rate Using a Control for Health Expenditure, 2000-2019 | | | | | |
| --- | --- | --- | --- | --- | --- |
|  | Model 1 | Model 2 | Model 3 | Model 4 |  |
|  | Without imputation | With imputation | Without imputation | With imputation |  |
| Abortion policy index 1 | -0.001 | 0.027 |  |  |  |
|  | (0.024) | (0.025) |  |  |  |
| Abortion policy index 3 |  |  | -0.138 | 0.033 |  |
|  |  |  | (0.106) | (0.162) |  |
| Control variables |  |  |  |  |  |
| Mean abortion policy index 1 in neighboring countries | -0.079 | 0.041 |  |  |  |
|  | (0.043) | (0.051) |  |  |  |
| Mean abortion policy index 3 in neighboring countries |  |  | -0.468 | 0.188 |  |
|  |  |  | (0.260) | (0.286) |  |
| GDP per capita logged | 0.191 | 0.361*** | 0.144 | 0.377*** |  |
|  | (0.129) | (0.100) | (0.117) | (0.100) |  |
| Ratio female/male labor force participation | 0.003 | 0.000 | 0.004 | 0.000 |  |
|  | (0.003) | (0.003) | (0.002) | (0.002) |  |
| Infant mortality logged | -0.231 | 0.068 | -0.250 | 0.082 |  |
|  | (0.122) | (0.124) | (0.131) | (0.126) |  |
| Percent urban population | -0.020* | -0.015* | -0.019* | -0.015* |  |
|  | (0.009) | (0.006) | (0.008) | (0.006) |  |
| Median age of the population | 0.042* | -0.024 | 0.032 | -0.026 |  |
|  | (0.019) | (0.015) | (0.019) | (0.015) |  |
| Physicians per capita logged | -0.107 | -0.222** | -0.077 | -0.222** |  |
|  | (0.056) | (0.076) | (0.052) | (0.077) |  |
| Average years of women’s education | -0.296** | -0.465*** | -0.334** | -0.463*** |  |
|  | (0.098) | (0.066) | (0.103) | (0.067) |  |
| Contraceptive use | -0.029*** | -0.032*** | -0.029*** | -0.032*** |  |
|  | (0.005) | (0.004) | (0.005) | (0.004) |  |
| Women civil liberty index | -0.570* | -0.283 | -0.583* | -0.265 |  |
|  | (0.260) | (0.275) | (0.243) | (0.271) |  |
| Public health expenditure | 0.048* | 0.114*** | 0.048* | 0.116*** |  |
|  | (0.021) | (0.029) | (0.020) | (0.029) |  |
| Country FE | Yes | Yes | Yes | Yes |  |
| Year FE | Yes | Yes | Yes | Yes |  |
| Country-specific linear time trend | No | No | No | No |  |
| Constant | 6.560*** | 6.303*** | 7.349*** | 6.187*** |  |
|  | (1.504) | (1.079) | (1.530) | (1.090) |  |
| N/Countries | 2,576 | 8,175 | 2,190 | 8,175 |  |
|  | 145 | 185 | 144 | 185 |  |

Note: Standard errors in parentheses; ^*^ *p* < .05, ^**^ *p* < .01, ^***^ *p* < .001. Robust standard errors are clustered at the country level. In this test we do not include *abortion policy index 2* because the data on health expenditure and abortion policy index 2 overlap in very few years and in model without imputation the effect cannot be estimated.

Figure A2. Difference-in-difference effects of change in abortion law on TFR


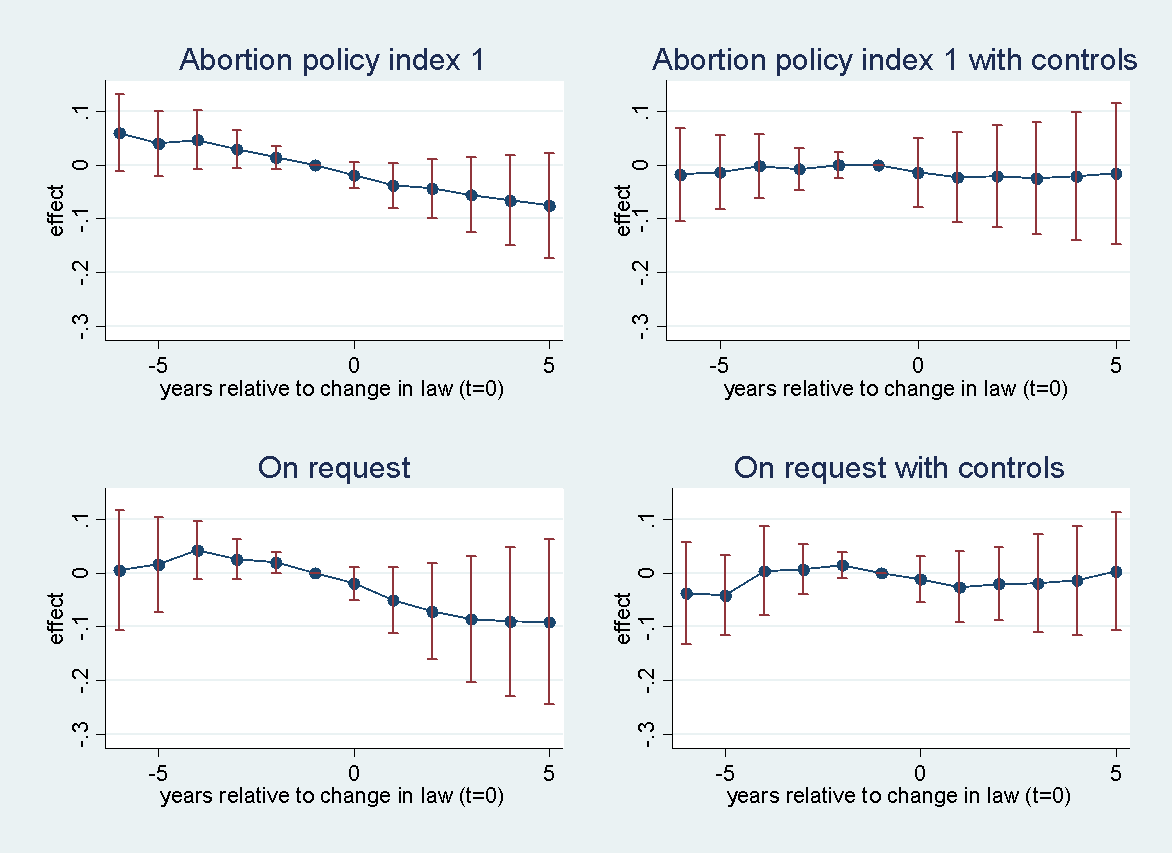


Notes: Graphs show the estimated instantaneous and dynamic (five years before and five years after)

treatment effects of abortion law liberalization on the TFR and placebo estimates. The upper two panels use *abortion policy index 1* (a discrete variable) as treatment. In the lower two panels we use as

“treatment” the change in legislation to allowing abortion “on request” (a binary treatment), and we

remove countries that have re-criminalized abortion (Hungary, Bahrain, Poland), to fit the staggered treatment design. Panels 1 and 3 to the left show the effect without adding control variables, while panels 2 and 4 to the right show the results after controlling for all socio-economic and political variables, and geographic lags.

| Table A11. FE Models Predicting the Fertility Rate Splitting by the Level of the Agricultural Labor Force without imputation, 1991-2019 | | | | | | | |
| --- | --- | --- | --- | --- | --- | --- | --- |
|  | Model 1 | Model 2 | Model 3 | Model 4 | Model 5 | Model 6 |  |
|  | Small agric. labor force | Large agric. labor force | Small agric. labor force | Large agric. labor force | Small agric. labor force | Large agric. labor force |  |
| Abortion policy index 1 | -0.025 | 0.014 |  |  |  |  |  |
|  | (0.028) | (0.020) |  |  |  |  |  |
| Abortion policy index 2 |  |  | -0.011 | 0.000 |  |  |  |
|  |  |  | (0.018) | (0.018) |  |  |  |
| Abortion policy index 3 |  |  |  |  | 0.025 | -0.417** |  |
|  |  |  |  |  | (0.115) | (0.128) |  |
| Control variables |  |  |  |  |  |  |  |
| Mean abortion policy index 1 in neighboring countries | -0.069 | 0.034 |  |  |  |  |  |
|  | (0.049) | (0.045) |  |  |  |  |  |
| Mean abortion policy index 2 in neighboring countries |  |  | -0.010 | 0.008 |  |  |  |
|  |  |  | (0.052) | (0.034) |  |  |  |
| Mean abortion policy index 3 in neighboring countries |  |  |  |  | -0.079 | 0.215 |  |
|  |  |  |  |  | (0.310) | (0.269) |  |
| GDP per capita logged | 0.563* | 0.253* | 0.595** | 0.270* | 0.598** | 0.022 |  |
|  | (0.223) | (0.112) | (0.197) | (0.106) | (0.213) | (0.121) |  |
| Ratio female/male labor force participation | -0.004 | -0.000 | 0.002 | -0.001 | -0.005 | 0.000 |  |
|  | (0.006) | (0.002) | (0.004) | (0.002) | (0.006) | (0.002) |  |
| Infant mortality logged | -0.053 | 0.139 | -0.004 | 0.044 | -0.081 | 0.136 |  |
|  | (0.136) | (0.132) | (0.171) | (0.127) | (0.142) | (0.193) |  |
| Percent urban population | -0.016 | -0.008 | -0.013 | -0.010 | -0.013 | -0.011 |  |
|  | (0.009) | (0.009) | (0.011) | (0.010) | (0.009) | (0.011) |  |
| Median age of the population | 0.009 | -0.032 | 0.012 | -0.057* | 0.005 | 0.006 |  |
|  | (0.024) | (0.022) | (0.031) | (0.024) | (0.027) | (0.025) |  |
| Physicians per capita logged | -0.333*** | -0.103* | -0.308** | -0.101 | -0.288*** | 0.006 |  |
|  | (0.085) | (0.045) | (0.103) | (0.053) | (0.079) | (0.051) |  |
| Average years of women’s education | -0.289* | -0.396*** | -0.614*** | -0.424*** | -0.384** | -0.365** |  |
|  | (0.135) | (0.092) | (0.175) | (0.105) | (0.133) | (0.108) |  |
| Contraceptive use | -0.016* | -0.035*** | -0.021** | -0.035*** | -0.015* | -0.030*** |  |
|  | (0.007) | (0.004) | (0.007) | (0.006) | (0.007) | (0.005) |  |
| Women civil liberty index | -0.572 | -0.276 | -0.620* | -0.106 | -0.715 | -0.501 |  |
|  | (0.371) | (0.209) | (0.249) | (0.169) | (0.432) | (0.279) |  |
| Country FE | Yes | Yes | Yes | Yes | Yes | Yes |  |
| Year FE | Yes | Yes | Yes | Yes | Yes | Yes |  |
| Country-specific linear time trend | No | No | No | No | No | No |  |
| Constant | 4.310 | 6.379*** | 5.867* | 7.283*** | 4.398 | 7.175*** |  |
|  | (2.312) | (1.171) | (2.525) | (1.186) | (2.413) | (1.500) |  |
| N/Countries | 2,011 | 3,318 | 1,035 | 2,719 | 1,700 | 1,482 |  |
|  | 93 | 128 | 76 | 122 | 88 | 79 |  |

Note: Standard errors in parentheses; ^*^ *p* < .05, ^**^ *p* < .01, ^***^ *p* < .001. Robust standard errors are clustered at the country level. Small (large) agricultural labor force is defined as have less (more) than 30.7% of workers in the agricultural sector. This is the average value in the database during the period.

| Table A12. FE Models Predicting the Fertility Rate Splitting by the Level of the Agricultural Labor Force with imputation, 1991-2019 | | | | | | | |
| --- | --- | --- | --- | --- | --- | --- | --- |
|  | Model 1 | Model 2 | Model 3 | Model 4 | Model 5 | Model 6 |  |
|  | Small agric. labor force | Large agric. labor force | Small agric. labor force | Large agric. labor force | Small agric. labor force | Large agric. labor force |  |
| Abortion policy index 1 | -0.016 | -0.010 |  |  |  |  |  |
|  | (0.024) | (0.036) |  |  |  |  |  |
| Abortion policy index 2 |  |  | 0.010 | -0.050* |  |  |  |
|  |  |  | (0.020) | (0.024) |  |  |  |
| Abortion policy index 3 |  |  |  |  | 0.107 | -0.434** |  |
|  |  |  |  |  | (0.110) | (0.130) |  |
| Control variables |  |  |  |  |  |  |  |
| Mean abortion policy index 1 in neighboring countries | -0.045 | 0.031 |  |  |  |  |  |
|  | (0.049) | (0.053) |  |  |  |  |  |
| Mean abortion policy index 2 in neighboring countries |  |  | 0.063 | 0.035 |  |  |  |
|  |  |  | (0.033) | (0.036) |  |  |  |
| Mean abortion policy index 3 in neighboring countries |  |  |  |  | -0.125 | -0.127 |  |
|  |  |  |  |  | (0.326) | (0.270) |  |
| GDP per capita logged | 0.657*** | 0.108 | 0.582*** | 0.078 | 0.611*** | 0.094 |  |
|  | (0.144) | (0.080) | (0.160) | (0.062) | (0.156) | (0.066) |  |
| Ratio female/male labor force participation | -0.004 | 0.002 | -0.004 | 0.000 | -0.004 | 0.000 |  |
|  | (0.005) | (0.002) | (0.005) | (0.002) | (0.005) | (0.002) |  |
| Infant mortality logged | -0.071 | 0.165 | -0.163 | 0.183 | -0.149 | 0.179 |  |
|  | (0.132) | (0.203) | (0.155) | (0.155) | (0.146) | (0.180) |  |
| Percent urban population | -0.007 | -0.016 | -0.001 | -0.011 | -0.005 | -0.012 |  |
|  | (0.008) | (0.009) | (0.009) | (0.010) | (0.008) | (0.009) |  |
| Median age of the population | -0.001 | -0.007 | -0.008 | -0.027 | -0.000 | -0.011 |  |
|  | (0.023) | (0.023) | (0.034) | (0.027) | (0.028) | (0.023) |  |
| Physicians per capita logged | -0.336*** | -0.064 | -0.344** | 0.012 | -0.346** | -0.021 |  |
|  | (0.092) | (0.056) | (0.123) | (0.052) | (0.111) | (0.050) |  |
| Average years of women’s education | -0.351** | -0.228* | -0.509*** | -0.153 | -0.424*** | -0.183 |  |
|  | (0.105) | (0.110) | (0.132) | (0.102) | (0.113) | (0.102) |  |
| Contraceptive use | -0.013* | -0.031*** | -0.015* | -0.028*** | -0.014* | -0.030*** |  |
|  | (0.006) | (0.005) | (0.006) | (0.006) | (0.006) | (0.005) |  |
| Women civil liberty index | -0.509 | -0.492 | -0.662* | -0.380 | -0.683* | -0.487 |  |
|  | (0.338) | (0.295) | (0.311) | (0.248) | (0.339) | (0.270) |  |
| Country FE | Yes | Yes | Yes | Yes | Yes | Yes |  |
| Year FE | Yes | Yes | Yes | Yes | Yes | Yes |  |
| Country-specific linear time trend | No | No | No | No | No | No |  |
| Constant | 3.650* | 6.344*** | 5.298** | 6.177*** | 4.680* | 6.286*** |  |
|  | (1.643) | (1.349) | (2.010) | (1.141) | (1.855) | (1.214) |  |
| N | 2,688 | 2,121 | 1,828 | 1,637 | 2,243 | 1,894 |  |
| Countries | 116 | 89 | 104 | 89 | 109 | 89 |  |

Note: Standard errors in parentheses; ^*^ *p* < .05, ^**^ *p* < .01, ^***^ *p* < .001. Robust standard errors are clustered at the country level. Small (large) agricultural labor force is defined as have less (more) than 30.7% of workers in the agricultural sector. This is the average value in the database during the period.

| Table A13. FE Models Predicting the Percent of Childless women and the TFR for the same sample of country years | | | | | | | |
| --- | --- | --- | --- | --- | --- | --- | --- |
|  | Model 1 | Model 2 | Model 3 | Model 4 | Model 5 | Model 6 |  |
|  | Perc. childless | Perc. childless | Perc. childless | TFR | TFR | TFR |  |
| Abortion policy index 1 | -0.062 |  |  | -0.018 |  |  |  |
|  | (0.225) |  |  | (0.020) |  |  |  |
| Abortion policy index 2 |  | 0.075 |  |  | -0.020 |  |  |
|  |  | (0.196) |  |  | (0.022) |  |  |
| Abortion policy index 3 |  |  | 1.935 |  |  | -0.278 |  |
|  |  |  | (5.924) |  |  | (0.491) |  |
| Control variables |  |  |  |  |  |  |  |
| Mean abortion policy index 1 in neighboring countries | 1.927** |  |  | -0.092 |  |  |  |
|  | (0.613) |  |  | (0.057) |  |  |  |
| Mean abortion policy index 2 in neighboring countries |  | 1.253 |  |  | -0.117* |  |  |
|  |  | (0.608) |  |  | (0.052) |  |  |
| Mean abortion policy index 3 in neighboring countries |  |  | 7.076 |  |  | 0.324 |  |
|  |  |  | (9.359) |  |  | (0.415) |  |
| GDP per capita logged | 1.505 | 2.996 | -0.629 | 0.151 | 0.103 | -0.040 |  |
|  | (3.451) | (3.916) | (3.447) | (0.184) | (0.238) | (0.164) |  |
| Ratio female/male labor force participation | 0.023 | 0.022 | -0.088 | 0.012* | 0.014* | 0.008 |  |
|  | (0.093) | (0.084) | (0.138) | (0.005) | (0.006) | (0.005) |  |
| Infant mortality logged | -1.101 | -0.133 | -1.309 | 0.245 | 0.357 | 0.050 |  |
|  | (2.505) | (2.395) | (2.318) | (0.169) | (0.187) | (0.130) |  |
| Percent urban population | -0.301 | -0.377* | -0.273 | 0.001 | 0.001 | 0.001 |  |
|  | (0.153) | (0.152) | (0.226) | (0.005) | (0.007) | (0.007) |  |
| Median age of the population | 1.504** | 1.697** | 2.259** | -0.031 | -0.046 | -0.039 |  |
|  | (0.452) | (0.505) | (0.735) | (0.036) | (0.043) | (0.033) |  |
| Physicians per capita logged | 0.730 | 2.310 | -1.334 | -0.148 | -0.184 | -0.143 |  |
|  | (2.547) | (2.430) | (2.260) | (0.199) | (0.198) | (0.162) |  |
| Average years of women’s education | -2.399 | -3.331* | 1.701 | -0.139 | -0.111 | -0.033 |  |
|  | (1.795) | (1.427) | (3.548) | (0.129) | (0.131) | (0.104) |  |
| Contraceptive use | -0.154 | -0.110 | -0.254 | -0.001 | 0.000 | 0.007 |  |
|  | (0.077) | (0.065) | (0.173) | (0.004) | (0.004) | (0.005) |  |
| Women civil liberty index | -3.160 | -4.958 | -1.668 | -0.617* | -0.601* | 1.611 |  |
|  | (3.390) | (2.614) | (31.886) | (0.223) | (0.227) | (0.931) |  |
| Country FE | Yes | Yes | Yes | Yes | Yes | Yes |  |
| Year FE | Yes | Yes | Yes | Yes | Yes | Yes |  |
| Country-specific linear time trend | No | No | No | No | No | No |  |
| Constant | -8.503 | -25.229 | -37.809 | 3.177 | 3.665 | 1.679 |  |
|  | (32.676) | (36.066) | (51.255) | (2.287) | (2.937) | (2.683) |  |
| N | 157 | 141 | 114 | 157 | 141 | 114 |  |
| Countries | 24 | 24 | 23 | 24 | 24 | 23 |  |

| Table A14. FE Models Predicting the Fertility Rate Using Controls for Religiosity, 1970-2010 | | |
| --- | --- | --- |
|  | Model 1 | Model 2 |
|  | Without imputation | With imputation |
| On request | 0.098 | -0.066 |
|  | (0.091) | (0.103) |
| Control variables |  |  |
| Mean on request in neighboring countries | 0.546* | 0.782** |
|  | (0.249) | (0.242) |
| GDP per capita logged | 0.502*** | 0.300** |
|  | (0.116) | (0.093) |
| Ratio female/male labor force participation | 0.003 | 0.000 |
|  | (0.003) | (0.002) |
| Infant mortality logged | -0.094 | 0.110 |
|  | (0.148) | (0.140) |
| Percent urban population | -0.012 | -0.012 |
|  | (0.008) | (0.006) |
| Median age of the population | -0.006 | -0.021 |
|  | (0.022) | (0.019) |
| Physicians per capita logged | -0.198** | -0.128 |
|  | (0.066) | (0.082) |
| Average years of women’s education | -0.581*** | -0.511*** |
|  | (0.088) | (0.071) |
| Contraceptive use | -0.036*** | -0.035*** |
|  | (0.005) | (0.005) |
| Women civil liberty index | -0.163 | -0.178 |
|  | (0.226) | (0.233) |
| Percent Catholic | -1.288 | -1.282* |
|  | (0.715) | (0.570) |
| Percent Muslim | 1.895 | 1.794* |
|  | (1.218) | (0.692) |
| Country FE | Yes | Yes |
| Year FE | Yes | Yes |
| Country-specific linear time trend | No | No |
| Constant | 6.096*** | 6.574*** |
|  | (1.344) | (1.109) |
| N | 4,191 | 6,537 |
| Countries | 143 | 184 |

Note: Standard errors in parentheses; ^*^ *p* < .05, ^**^ *p* < .01, ^***^ *p* < .001. Robust standard errors are clustered at the country level.

*Definition and sources of each variable*

*Total fertility rate* (TFR): average number of children women are expected to bear through their entire fertile years given the current-age specific birth rates. Data for this variable originates from the United Nations’ (2019).

*Abortion policy index 1*: The database contains yearly information on the legal status of six grounds to conduct an (1) *life risks* of the pregnant woman, (2) *health risks*, (3) *rape*, (4) *fetal* *impairment*, (5) *socio*-*economic* conditions and (6) *on request*. *Life risks* refers to the situation when the pregnancy can be medically considered life-threatening. *Health risks* is understood as the condition when the continuation of pregnancy would involve an injury or clear harm for the physical or mental health of the pregnant woman. *Rape* refers to situations when the pregnancy is the result of sexual violence. *Fetal impairment* is considered when the pregnancy is not medically viable or the fetus has been diagnosed with a major handicap. *Socio-economic* grounds refer to the situation in which the pregnant woman has social characteristics (e.g. number of children) or a foreseeable environment (e.g. poverty) that prevents adequate child raising. *On request* grounds refer to the situation where the woman’s decision to have an abortion suffices and requires no approval from a medical committee to access this surgical procedure. By definition, it entails legalization *on request* involves the legalization of all other five grounds.

Each of these six variables are dichotomous time changing. If the ground was initially illegal it is coded as 0 until the legalization occurs and in that year shifts to 1. If the ground is recriminalized again, the value returns to 0 in the following year until another possible decriminalization occurs. The final database covers 195 independent states and the period 1950-2015.

The database of abortion policy was constructed combining multiple sources: major cross-national reviews of abortion policy (Rowlands 2012; United Nations 2001; World Health Organization Several years; World Health Organization 2019); concrete statutes; and, whenever possible three case studies of abortion policy history or specific reform in each country. The legal regime and each legal reform of each of the 195 countries was entered – either verbatim or as summarized in the original source –in a master file. As a general principle, each ground is considered legal only fi expressly enshrined in the law. Liberalizations are defined as legal reforms that increase the number of grounds on which abortion is legal. If the country recriminalized that ground again, that condition is reclassified for the following year onwards as not legal. For countries where abortion is determined at the subnational level, we take the legal regime of the state or province with the largest population (Frank et al 2010).

*Abortion policy index 2*: Data from Finlay et al. (2013).

*Abortion policy index 3*: Data obtained from Teorell et al. (2020).

*Mean value in abortion policy index 1 in neighboring countries*: Average value in *abortion policy index 1* in neighboring countries in that year. Neighboring countries defined as separated by land or river border or less than 400 miles of water. The contiguity dataset is from Stinnett et al. (2017).

*Mean value in abortion policy index 2 in neighboring countries*: The same procedure as with mean value in *abortion policy index 1* in neighboring countries but using *abortion policy index 2.*

*Mean value in abortion policy index 3 in neighboring countries*: The same procedure as with mean value in *abortion policy index 1* in neighboring countries but using *abortion policy index 3.*

*Percent of neighboring countries that have liberalized abortion on request:* The sample procedure as with *Mean value in abortion policy index 1 in neighboring countries* but using the variable *on request* from *abortion policy index 1.*

*Average years of women’s education*: Average years of education of women 15 or older. Data are from Wittgenstein Centre for Demography and Global Human Capital (2018).

*Infant mortality rate*: Mortality rate, under-1 (per 1,000 live births). Data are from World Bank (2020).

*Female labor force participation ratio* Ratio of female labor force participation/male labor force participation. The indicator is the Labor force participation rate, female (% of female population ages 15+) (national estimate). Data are from World Bank (2022).

*Contraceptive prevalence*: Median estimate of the percentage of married women 15-49 that use modern contraceptive methods. We select the value of married women because it produces the smallest number of missing values. Data are from United Nations (2020).

*GDP per capita*: PPP-based GDP per capita in 2005. Data are from James (2012).

*Physicians per capita*: Rate of physicians per 1,000 individuals. Data are from World Bank (2020) and Banks et al. (2020).

*Urban population*: Percentage of Population at Mid-Year Residing in Urban Areas by region. Data are from United Nations (2020).

*Women civil liberty index:* The index is formed by taking the point estimates from a Bayesian factor analysis model of the indicators for freedom of domestic movement for women (v2cldmovew), freedom from forced labor for women (v2clslavef), property rights for women (v2clprptyw), and access to justice for women (v2clacjstw) (Coppedge et al. 2021).

*Total fertility rates* of different age groups: Data are from United Nations (2022).

*Adjusted fertility rate:* Obtained from Jdanov et al. (2022).

*Percent childless women:* Data are from OECD (2022).

*Percent Catholics:* Data are from Maoz and Henderson (2013).

*Percent Muslims:* Data are from Maoz and Henderson (2013).

*Health expenditure:* Domestic general government health expenditure (% of GDP). Data are from World Bank (2022).

*Agricultural labor force:* Agricultural workers as a percent of the labor force. Data are from World Bank (2022).

*Sources*

Banks A, et al., Cross-National Time-Series Data Archive. Databanks International. (2012). Jerusalem.

Coppedge, M., J. Gerring, CH Knutsen. (2021). V-Dem [Country–Year/Country–Date] Dataset v11.1. Varieties of Democracy (V-Dem) Project.

David E., Canning, D., Fink, G. & Finlay, J. (2009). Fertility, Female Labor Force Participation, and the Demographic Dividend, *Journal of Economic Growth*, *14*(2), 79-101

Finlay, Jocelyn; Canning, David; Po, June, 2013, "Reproductive Health Laws Around the World 1960-present", <https://doi.org/10.7910/DVN/1WKMXK>, Harvard Dataverse, V3

Jdanov, D. et al. (2022). Human Fertility Database. Max Planck Institute for Demographic Research.

Maoz Z & Henderson, E. A. (2013). The World Religion Dataset, 1945-2010: Logic, Estimates, and Trends. *International Interactions*, *39*, 265-291.

OECD. (2022). OECD Family Database. OECD.

Rowlands, S. (2012). Abortion Law of Jurisdictions around the World, International Federation of Professional Abortion and Contraception Associates.

Stinnett, DM, Tir, J., Schafer, P., Diehl, P. F., & Gochman, C. (2002). The Correlates of War Project Direct Contiguity Data, Version 3. *Conflict Management and Peace Science 19* (2):58-66.

Teorell, J.. et al. (2020). The Quality of Government Standard Dataset, 2020. The Quality of Government Institute.

United Nations. (2022) *World Population Prospects 2022*. United Nations.

United Nations. (2007). *Abortion policies: A global review*. Department of Economic and Social Affairs, Population Division.

United Nations. (2018). *World Urbanization Prospects 2018*. United Nations.

United Nations. (2020). *Estimates and projections of Family Planning Indicators*, 2020. United Nations.

Wittgenstein Centre for Demography and Global Human Capital. (2018). *Wittgenstein Centre Data Explorer Version 2.0 (Beta)*.

World Bank. (2022). *World Development Indicators*. World Bank.

World Health Organization. (2019). *Global Abortion Policies Database*. World Health Organization.

World Health Organization. (several years). *International Digest of Health Legislation*. World Health Organization.
